# Characterization of Gene Expression Associated with Drought Avoidance and Tolerance Traits in a Perennial Grass Species

**DOI:** 10.1371/journal.pone.0103611

**Published:** 2014-08-25

**Authors:** Peng Zhou, Yuan An, Zhaolong Wang, Hongmei Du, Bingru Huang

**Affiliations:** 1 School of Agriculture and Biology, Shanghai Jiao Tong University, Shanghai, P. R. China; 2 Department of Plant Biology and Pathology, Rutgers, the State University of New Jersey, New Brunswick, New Jersey, United States of America; Key Laboratory of Horticultural Plant Biology (MOE), China

## Abstract

To understand molecular mechanisms of perennial grass adaptation to drought stress, genes associated with drought avoidance or tolerance traits were identified and their expression patterns were characterized in C4 hybrid bermudagrass [Cynodon dactylon (L.) Pers.×C. transvaalensis Burtt Davy, cv. Tifway] and common bermudagrass (C. dactylon, cv. C299). Plants of drought-tolerant ‘Tifway’ and drought-sensitive ‘C299’ were exposed to drought for 5 d (mild stress) and 10 d (severe stress) by withholding irrigation in a growth chamber. ‘Tifway’ maintained significantly lower electrolyte leakage and higher relative water content than ‘C299’ at both 5 and 10 d of drought stress. Four cDNA libraries via suppression subtractive hybridization analysis were constructed and identified 277 drought-responsive genes in the two genotypes at 5 and 10 d of drought stress, which were mainly classified into the functional categories of stress defense, metabolism, osmoregulation, membrane system, signal and regulator, structural protein, protein synthesis and degradation, and energy metabolism. Quantitative-PCR analysis confirmed the expression of 36 drought up-regulated genes that were more highly expressed in drought-tolerant ‘Tifway’ than drought-sensitive ‘C299’, including those for drought avoidance traits, such as cuticle wax formation (CER1 and sterol desaturase), for drought tolerance traits, such as dehydration-protective proteins (dehydrins, HVA-22-like protein) and oxidative stress defense (superoxide dismutase, dehydroascorbate reductase, 2-Cys peroxiredoxins), and for stress signaling (EREBP-4 like protein and WRKY transcription factor). The results suggest that the expression of genes for stress signaling, cuticle wax accumulation, antioxidant defense, and dehydration-protective protein accumulation could be critically important for warm-season perennial grass adaptation to long-term drought stress.

## Introduction

With the decline in water resources and the increase in human demand for water, water for irrigation is becoming increasingly limited. Drought stress is becoming a significant abiotic stress limiting plant growth and production in many areas. Plants develop various stress resistance mechanisms involving avoidance and tolerance strategies, which vary with plant species, duration and severity of the stress [1]. Dehydration avoidance of leaves is characterized by reducing water loss through mechanisms, such as stomatal closure and accumulation of wax on leaf surfaces while dehydration or desiccation tolerance has been associated with traits, such as osmotic adjustment, sugar accumulation, and maintenance of the integrity of membranes and proteins from dehydration damage [1]–[3].

At the molecular level, various studies identified drought-regulated genes in different plant species, mostly in annual crop plants and the model plant *Arabidopsis* [4], [5]. For example, using cDNA microarray analysis in barley (*Hordeum vulgare* L. cv. Tokak), Ozturk et al. [6] identified significant up-regulation of jasmonate-responsive, metallothionein-like, late-embryogenesis-abundant (LEA) and ABA-responsive proteins and down-regulation of genes for photosynthesis under short-term (6 and 10 h) drought stress. Seki et al. [7] reported that 44 full-length cDNA clones were modulated by 2 fold or greater at the mRNA level in *Arabidopsis* exposed to 2 h of drought stress, including LEA 76 type 1 protein, a nonspecific lipid transfer protein, a putative water channel protein, and HVA22 homolog. In alfalfa (*Medicago sativa*) exposed to 3- and 8-h drought stress, Chen et al. [8] identified some drought-responsive genes, such as two heat shock-related protein genes, dehydrin and xyloglucan endotransglycosylase. Xue et al. [9]reported by using quantitative RT-PCR and cDNA microarray, genes encoding chloroplast enzymes involved in carbon fixation were down-regulated in wheat (*Triticum aestivum*) leaves exposed to a prolonged period (7 d) of drought stress, while those encoding cytoplasmic and vacuolar enzymes in the pathways leading to glucose, fructose and fructan production were up-regulated. Previous studies have demonstrated that drought-regulated genes and their expression patterns varied with plant species and stress duration or severity. However, knowledge of drought-regulated gene expression patterns associated with intraspecific genetic variation and temporal regulation is generally limited in perennial grass species, which often endure long-term drought stress in natural environments. Such information is important for developing effective engineering strategies to improve stress tolerance.

Dehydration avoidance or tolerance to mild or moderate drought level is important for maintaining grain yield production for annual crops, whereas survival mechanisms through long-term or severe drought is critical for perennial grass species due to their perennial nature. In a resurrection grass species, *Sporobolus stapfianus*, Blomstedt et al. [10] identified some unique genes encoding abundant drought-induced proteins or low-abundance transcripts that were not previously found in other species by differential screening. Using comparative analysis between desiccation-tolerant *S. stapfianus* and desiccation-sensitive grass *S. pyramidalis*, Gaff et al. [11] found 12 novel proteins associated with desiccation tolerance in the tolerant grass species. Perennial forage grasses and turfgrass species are cultivated in many areas with limited irrigation. Many cultivated perennial grass species, such as bermudagrass (*Cynodon* spp.), exhibit a wide range of genetic variation, which is a valuable germplasm for studying drought tolerance mechanisms in warm-season perennial grasses [12]–[15]. Triploid hybrid bermudagrass (*Cynodon dactylon*×*C. transvaalensis*) has been developed to produce highly desirable turf quality with limited irrigation [13], which exhibited better drought tolerance than common bermudagrass [*C. dactylon* (L.) Pers.] due to greater maintenance of photosynthetic processes, water status, and antioxidant defenses [16]–[18]. The identification of genes associated with genetic variation in drought tolerance in bermudagrass in response to short-term, mild drought and long-term, severe drought will provide further insight into the molecular basis for drought tolerance in perennial grass species.

The objectives of this study were to identify up-regulated genes in bermudagrass in response to short-term or mild drought stress (withholding irrigation for 5 d) and long-term, severe drought stress (withholding irrigation for 10 d) and to compare differentially-expressed genes between a drought-tolerant hybrid bermudagrass genotype (‘Tifway’) and a drought-sensitive common bermudagrass genotype (‘C299’) using the subtraction suppression hybridization (SSH) technique. SSH has successfully been used to identify genes responsive to various biotic and abiotic stresses in various plant species [19]–[21]. Expression of selected genes from SSH libraries were confirmed using quantitative RT-PCR, and major metabolic processes and pathways regulating bermudagrass adaptation to mild and severe drought were discussed using the functional analysis of known protein sequences through BLAST searches.

## Materials and Methods

### Plant materials, growing conditions, and treatments

Plants of a drought-tolerant genotype (‘Tifway’ or Tifway) of hybrid bermudagrass (*C. dactylon* L.×*C. transvaalensis* L.) and a drought-sensitive genotype (‘C299’) of common bermudagrass (*C. dactylon*) were collected from 3-year-old field plots at the research farm in Shanghai Jiao Tong University, Shanghai, China. Plants (approximately 50) were vegetatively propagated in each plastic pot (15-cm diameter and 14 cm in depth) filled with a mixture (1∶3, v/v) of sand and sandy loamy soil (fine-loamy, mixed mesic Typic Hapludult). Plants were maintained in a growth chamber with a temperature regime of 30/25°C (day/night), a 14-h photoperiod, 75% relative humidity, and a photosynthetically active radiation of 480 µmol m^−2^ s^−1^ at the canopy level. Plants were irrigated three times per week until soil water reached field capacity or drainage occurred from the bottom of the pot and fertilized biweekly with controlled-release fertilizers (15 N-15 P_2_O_5_-10 K_2_O) at a total amount of 57 kg N ha^−1^. Plants were maintained in the above conditions for 40 d to allow the establishment of shoot canopy and root systems.

The experiment consisted of three treatments. In the drought stress treatment, plants for each genotype were not watered for 5 d or 10 d. The control plants of both genotypes were watered thoroughly every other day until soil water content reached field capacity (drainage observed from the pot). Each treatment for each grass genotype had three replicates (pots). Two treatments and two genotypes were arranged randomly inside the growth chamber.

### Physiological analysis

Differences in drought tolerance between the two genotypes were characterized by whole plant physiological characteristics, including cell membrane stability and relative water content (RWC) of leaves. Cell membrane stability was determined as electrolyte leakage (EL) on 10 second and third fully-expanded leaves in each container. For EL analysis, 0.1 g fresh leaf segments (approximately 0.5 cm long) from each sample were incubated in 15 mL distilled deionized water on a shaker for 24 h. The conductance of the incubation solution was measured as the initial level of electrolyte leakage (Ci) using a conductance meter (YSI Model 32, YSI Incorporated, Yellow Springs, OH, USA). Leaf tissue in the incubation solution was killed in an autoclave at 120°C for 30 min. The conductance of the incubation solution with killed tissues (Cmax) was determined following 24-h incubation on a shaker. Relative EL was calculated as (Ci/Cmax)×100 [22].

Leaf RWC was determined using 10–15 second and third fully-expanded leaves in each container according to Barrs and Weatherley (1962). Leaf samples were detached from the plants and immediately weighed to determine fresh weight (FW). Samples were placed into covered petri dishes filled with water for leaves to reach full hydration. After approximately 24 h at 4°C, leaf samples were blotted dry with paper towels, and weighed to determine turgid weight (TW). Leaf tissue was then dried in an oven at 80°C for 48 h to determine dry weight (DW). Leaf RWC was calculated as: (FW-DW)/(TW-DW)×100.

### Construction of suppression subtractive hybridization (SSH) library

Fully-expanded leaves from each genotype exposed to the well-watered control, and at drought stress for 5 d and 10 d were collected for the extraction of total RNA and construction of the SSH library to identify responsive genes differentially expressed in the two genotypes. Total RNA was extracted using the Trizol Reagent (Invitrogen, Carlsbad, CA, USA). Poly (A_+_) RNA was isolated from total RNA using mRNA Oligotex isolation midi kit (Qiagen, Valencia, CA, USA). The subtraction library was prepared using PCR-Select cDNA subtraction kit (Takara BIO, Inc., Otsu, Shiga, Japan) according to the manufacturer's instructions. In order to identify drought up-regulated genes, the cDNAs generated from plants under drought stress for 5 or 10 d served as the testers, while the cDNAs generated from the well-watered control plants were used as the drivers. The tester and the driver cDNAs were synthesized separately from 2 µg poly (A_+_) RNA isolated from plants grown under normal or drought stress conditions. Both the tester and driver cDNAs were digested with *Rsa* I and then the tester cDNAs were divided into two portions, each ligated with two different cDNA adaptors (Adaptor 1: 5′- CTAATACGACTCACTATAGGGCTCGAGCGGCCGCCCGGGCAGGT -3′; Adaptor 2: 5′-CTAATACGACTCACTATAGGGCAGCGTGGTCGCGGCCGAGGT-3′). Two rounds of hybridization and PCR amplification were performed to enrich for the differentially-regulated gene sequences. The subtracted cDNA population was then subjected to primary PCR amplification of differentially-expressed genes using the adaptor specific primer (5′-CTAATACGACTCACTATAGGGC-3′). Subsequently, a second PCR amplification was performed with nested primers (Primer 1: 5′- TCGAGCGGCCGCCCGGGCAGGT-3′; Primer 2:5′-AGCGTGGTCGCGGCCGAGGT-3′) to reduce the background and to further enrich for differentially-expressed genes. After the second PCR, the products were directly cloned into the pMD18-T vector using TA Cloning kit (Takara BIO, Inc., Otsu, Shiga, JapanThe ligated product was transformed into *E. coli* competent cells (DH5α) which were then plated onto LB agar medium containing 100 µg ml^−1^ ampicillin and incubated at 37°C overnight. A total of 1302 positive clones were obtained from ‘C299’ and ‘Tifway’. Using PCR with nested primers, 803 clones were confirmed to have inserted cDNA fragment in these four SSH libraries. Each clone was picked individually and grown overnight in 1.0 mL liquid LB medium with 100 µg mL^−1^ ampicillin. The clones were stored as glycerol stocks at −80°C for sequencing.

### DNA sequencing and analysis

All 803 clones were sequenced by Invitrogen sequencing company (Shanghai China) and 757 sequences were obtained ranging in length from 88 to 823 bp. After removing contaminated vectors, mitochondrial, ribosomal, and *E.coli* sequences, 444 high quality ESTs were obtained and analyzed using the BlastN and BlastX algorithm (http://www.ncbi.nlm.nih.gov/BLAST) to identify their putative functions. All ESTs were submitted to genebank. Their accession numbers are from JK340473 to JK340916.

### Quantitative (Q)-PCR analysis

Total RNA was extracted as described above. The first strand cDNA was generated with a RETROscript Kit (Applied Biosystems/Ambion Inc.,Austin Tx) using oligo (dT) as primer. Thirty seven genes were detected by Quantitative (Q)-PCR. Q-PCR analysis was performed with specific primers as shown in Table 1 to detect the transcripts selected from the SSH library in both genotypes under the well-watered control and drought stress conditions. Q-PCR amplification mixtures (25 µl) contained 25 ng template cDNA, 2*SYBR Green I Master Mix buffer (12.5 ul)(Applied Biosystems) and 300 nM forward and reverse primer. Reactions were run on an ABI PRISM 5700 Sequence Detector (Applied Biosystems). The cycling conditions comprised 4 min polymerase activation at 94°C and 40 cycles at 94°C for 30 sec, 58°C for 30 sec and 72°C for 30 sec. 18S ribosomal RNA gene (primers in table 1, accession number: AB536694) was selected as endogenous control. Each gene was detected three times. All PCR efficiencies were above 95%. Sequence Detection Software (version 1.3) (Applied Biosystems) results were exported as tab-delimited text files and imported into Microsoft Excel for further analysis. The median coefficient of variation (based on calculated quantities) of duplicated samples was 6%.

**Table 1:** Primers sequences for RT-PCR.

Primers	Accession number	Forward sequence 5′-3′	Reverse sequence 5′-3′
18S	AB536694	GTGACGGGTGACGGAGAATT	GACACTAATGCGCCCGGTAT
CdL1	JK340473	ACTGGTTCAGAACTCTGCAA	ACCACATCGAAATGCATCAC
CdL2	JK340477	ACACTGTCATGCATGTTGGC	ACCTCCTGCACAGGCTTCTT
CdL4	JK340476	TAAAGACAGGCAGAGCCAGC	ACCCTTAAGCCCTACACGAA
CdL9	JK340478	ACTTTGAACCTTCACGGAGGCT	ACCGTGCATGTCAATGGTTGAT
CdL8	JK340518	ACACATACATACGTGATACGTCAA	TCCTGGACTACTGCGAATCT
CdL19	JK340483	ACTTCTCATAAATTCGTTGAAGGC	ACAGCCAGCATTTTATGAAGCTTT
CdL20	JK340484	ACCTCTGGTTGCACTCGTTG	TGTATGCATAGAGCCACACG
CdL35	JK340626	ACAAGCCTGGTGACACGATA	TACCCTACCACATTGACGGC
CdL36	JK340585	ACCTGTAGCCTTTATCTGTC	ATTGGCTCTGGTTTACCTTC
CdL37	JK340586	ACATTCCGACTGTGATGTCG	AGGTGATTGAGGCTATGTCC
CdL40	JK340589	GCTCACGGAAGCCCAGATTA	CCACGCTCATCCTTCTCCAT
CdL42	JK340615	ACGAACTGCCATAGAGGATC	ACTGGCTTTCCAGTCAAACG
CdL44	JK340578	TCCACGTCGTGCTTGTCCTT	CGTGAAGAGGAAGAAGAAGAAGGG
CdL49	JK340642	GGGAGAAGTAACAACGGATG	GCCGTTATCTCATCTCCAAT
CdL52	JK340628	CAAGTGACGGGCTATGGGAC	CGACCACGCAGGTTATGTTG
CdL56	JK340591	ACCAACCAAACTAAACCCAAGC	GCCTCTACGCTTCAGTTCAC
CdL61	JK340592	CGCCCAGAACCTTCTTCATATA	GGAGATCGTGAGCACATACT
CdL62	JK340580	TGCACAAGGAACACAGATCG	GTTGCAACTTGCCTAGGTTCA
CdL67	JK340607	GGTGTGGCTCCACGTCTTCT	ACGAGAACATCGACGAGCTG
CdL68	JK340608	GCAGAGAGAGTGAGAGGGCA	CGGAGCAGGAGTTCTACAGG
CdL69	JK340599	CTGAAGGAAGCCCTATGGTT	GTTGACGGGCTACTAACTAC
CdL74	JK340625	ACACGTATGGGTTTAGGTGGTG	TTCAAGAAGGAGCTGGACGG
CdL79	JK340618	CATCGTTGCGTTGCACCGTC	ACGGGCAGAAGCAAATTGAGG
CdL80	JK340610	TGCCAATACACAACACAAGAGC	CATCCACAAGGACGCAGAAG
CdL82	JK340582	ACCCCTTGTATGCGTCGGTG	CAGATCGGGATCCAGTAGATGAGG
CdL85	JK340595	CGACTAAGGAAGAACGGTCC	CAGGGATAAGACAGGAGCGA
CdL96	JK340598	ATCCTGTGCTTGCTCCTGGC	AGGTTCTTGACGAGCGGGAG
CdL111	JK340584	GAATCCGAGCACTCTCAGGA	CTCAAGACAACAGTCAGACACA
CdL113	JK340639	ATAGTTGAGATTCCAGGCTC	TCAACTGAGAAGGCTAAGAC
CdL10–27	JK340525	AGTTGTGGAAGGATGGAGAA	CCTGGGAAGGACTGATGAAG
CdL10–39	JK340529	AGGCTGCCCTGATGTGATTT	ATTGGAATGACTCGTGTCGC
CdL10–52	JK340708	AGCGAGCGACTTACAATGGC	AACACTGAAGGCGTGGCTGA
CdL10–99	JK340714	TTTGATCCCTGATCAGGGCA	GCCTGAAGAGTCCTCATGGT
CdL10–127	JK340747	GTTGGTGAGGCTTTCTGGGA	GTGGTGATGGCAAGTGGATTG
CdL10–97	JK340737	AGTCCACTGGTGTCTTCACT	GACACCAACAACGAACATGG
CdL10–47	JK340720	TCACGATGGAGGAGAACCAC	AGCATTTCCTTGTGGACGGG
Tifway-10–46	JK340856	ACCATACAGACCCTTCAGAC	GCGAACAGTCCGTGATAACT

### Hierarchical clustering analysis

Thirty seven drought-responsive genes confirmed with Q-PCR were subjected to clustering analysis. For comparison among treatments, let G_i_ equal the primary data for gene G in condition *i*. The transformation used was: g_i_ = (G_i_−G_average_)/G_average_, with G_average_ referring to the average expression quantity of gene G relative to the total expression. After transformation, g_i_ values were used for cluster analysis. Significantly expressed genes were hierarchically clustered with average linkage and Euclidean distance as a measurement of similarity using Genesis version 1.7.5 (Graz University of Technology, http://www.genome.tugraz.at). To display the clustering results, each gene in a certain condition was assigned a cell. Red, green, and black boxes represent genes that increased, decreased, and had equal expression levels at time points after withholding water, respectively. This allows for ease of visualization of the relationship of each gene and the expression patterns.

## Results

### Physiological responses of two bermudagrass genotypes to drought stress

Leaf relative water content (RWC) was maintained over 90% in both genotypes under well-watered conditions, but declined to 88% for ‘Tifway’ and 69% for ‘C299’ by 5 d of drought stress, and to 69% for ‘Tifway’ and 29% for ‘C299’ by 10 d of drought stress (Table 2). Leaf cell membrane stability was evaluated by measuring electrolyte leakage (EL). Leaf EL increased with drought stress, to 27% for ‘Tifway’ and 49% for ‘C299’ by 5 d of drought stress, and to 42% for ‘Tifway’ and 73% for ‘C299’ by 10 d of drought stress (Table 2). The differences in both RWC and EL between the drought-tolerant ‘Tifway’ and the drought-sensitive ‘C299’ and between treatments were statistically significant.

**Table 2:** Leaf relative water content (RWC) and electrolyte leakage (EL) of two bermugdagrass genotypes (‘Tifway’ and ‘C299’) at 5 and 10 d of drought stress and well-watered conditions.

Treatments	Genotypes	EL (%)	RWC (%)
Well watered	Tifway	23.7aA*	93.5aA
	C299	25.3cA	93.1aA
5-d drought	Tifway	27.2aB	88.0bA
	C299	48.6bA	69.4bB
10-d drought	Tifway	41.9bB	68.8cA
	C299	73.1aA	29.4cB

*Means within a column for either EL or RWC followed by the same letters were not significantly different based on LSD test at *P* = 0.05. Lower case letters for the comparison between treatments and uppercase letters for the comparison between two genotypes at a given treatment.

### Identification of genes up-regulated by drought stress in either genotype from the SSH libraries

Four SSH libraries were constructed to identify drought-up regulated genes in ‘C299’ and ‘Tifway’ in response to short-term (5 d) or mild drought and long-term (10 d) or severe of drought stress, as indicated by the levels of physiological responses described above.

**Figure 1:**
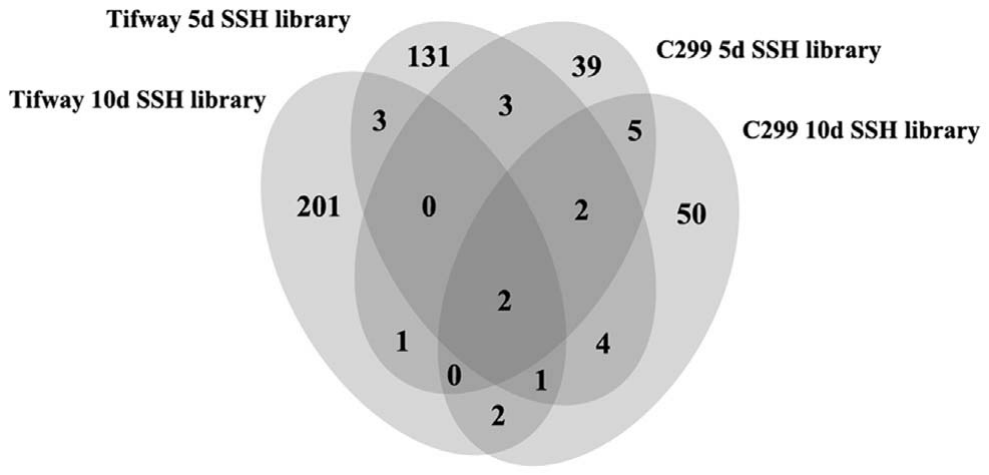
Four-way Venn diagram showing the number of genes differentially-expressed in ‘Tifway’ and ‘C299’ at 5 and 10 d of drought.

Using PCR with nested primers, 803 clones were confirmed to have inserted cDNA fragments from a total of 1272 individual clones in these SSH libraries. High throughput sequencing was conducted for all clones and with a 94% success rate. A total of 757 ESTs were generated with the length from 88 to 823 bp. After data assembly, 368 ESTs were found to be singlets, while the other 389 ESTs clustered into 76 contigs and each contig had 2–53 ESTs (Table 3). The ESTs generated from each library were also assembled and analyzed separately. With all contigs and singlets, 210 transcripts were identified in ‘Tifway’ at 10 d of drought stress, 145 in ‘Tifway’ at 5 d of drought stress, 66 in ‘C299’ at 10 d of drought stress, and 52 in ‘C299’ at 5 d of drought stress. In total, 421 genes were differentially expressed in at least one genotype at least one treatment duration period. The number of genes expressed in the two genotypes at the two different treatment periods is depicted with a four-way Venn diagram in Fig. 1.

**Table 3:** Summary of ESTs from bermudagrass drought stress SSH libraries.

Drought (days)	SSH clones	contigs	singlets	gene transcripts	contigs (refer to gene transcripts)
Tifway 10 d	230	24 (10.4%)	205 (89.6%)	210	5
5 d	196	74 (37.7%)	124 (62.3%)	145	21
C299 10 d	172	130 (75.6%)	42 (24.4%)	66	24
5 d	157	124 (79.0%)	33 (21.0%)	52	19
total	757	389(51.4%)	368(48.6%)	444	76

BLAST analysis of all the cDNA sequences was carried out to identify their putative functions (http://www.ncbi.nlm.nih.gov/BLAST). Sequences without significant homology with known proteins or genes were further analyzed with BLASTn using the EST others database (Supporting Information in File S1). About 84% ESTs had hit similarities in gene bank. Among them, 277 ESTs showed high homology to reported genes or proteins with E-value less than 1E-5. Of the 167 ESTs with no significant homology to reported genes or proteins, 105 were similar to reported ESTs, including one EST “Tifway-10–46” (Accession Number: JK340856) from ‘Tifway’-10-d-drought SSH library that had similarity to DHN4 gene encoding dehydrin protein. In the ‘C299’ SSH libraries, 25 clones expressed at 5-d drought and 41 clones expressed at 10-d drought had high homology to reported genes or proteins. In the ‘Tifway’ SSH libraries, 76 clones expressed at 5-d drought and 143 clones at 10-d drought had high homology to reported genes or proteins. Among these clones, many with known functions have also been demonstrated to be enhanced or induced by abiotic stress in other plant species, including *Arabidopsis thaliana*, maize (*Zea mays*) and rice (*Oryza sativa*).

### Functional classification of up-regulated genes in both bermudagrass genotypes under mild drought and severe drought stress

**Figure 2:**
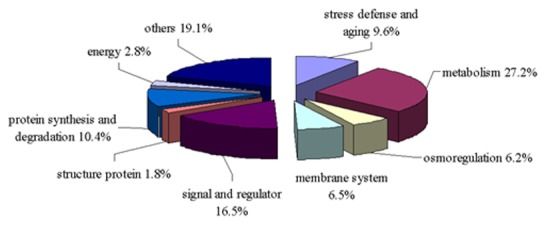
Classification of differentially-expressed genes between two genotypes at 5 and 10 d of drought. The differentially-expressed genes in ‘C299’ and ‘Tifway’ SSH libraries at 5 and 10 d of drought stress were grouped according to their putative functions generated by BlastX and BlastN analysis The percentage of genes in each functional group was listed.

In order to understand the molecular mechanisms underlying the adaptation of two bermudagrass genotypes differing in drought tolerance, up-regulated candidate genes isolated by SSH in response to short-term (5 d) and long-term (10 d) drought stress were categorized into various functional groups according to their main putative functions indicated by BLASTx and BLASTn analysis. Although some genes were involved in multiple metabolic processes, they were categorized according to their main functions in plant metabolism. A total of 277 gene transcripts with match to the GenBank database were grouped into nine functional categories: 1) stress defense and aging, 2) metabolism, 3) osmoregulation, 4) membrane system, 5) signal and regulator, 6) structure protein, 7) protein synthesis and degradation, 8) energy and 9) others which cannot be classified nor with unknown functions (Fig. 2). About 23% of gene transcripts annotated with unknown function were included in the category of unclassified proteins. The largest group of genes with known functions was metabolism-related genes and approximately 27.2% of the transcripts were included in this category, which was followed by signal and regulator (16.5%), protein synthesis and degradation (10.4%), stress defense and aging (9.6%), membrane system (6.5%), osmoregulation (6.2%), energy (2.8%), and structure protein (2%).

**Figure 3:**
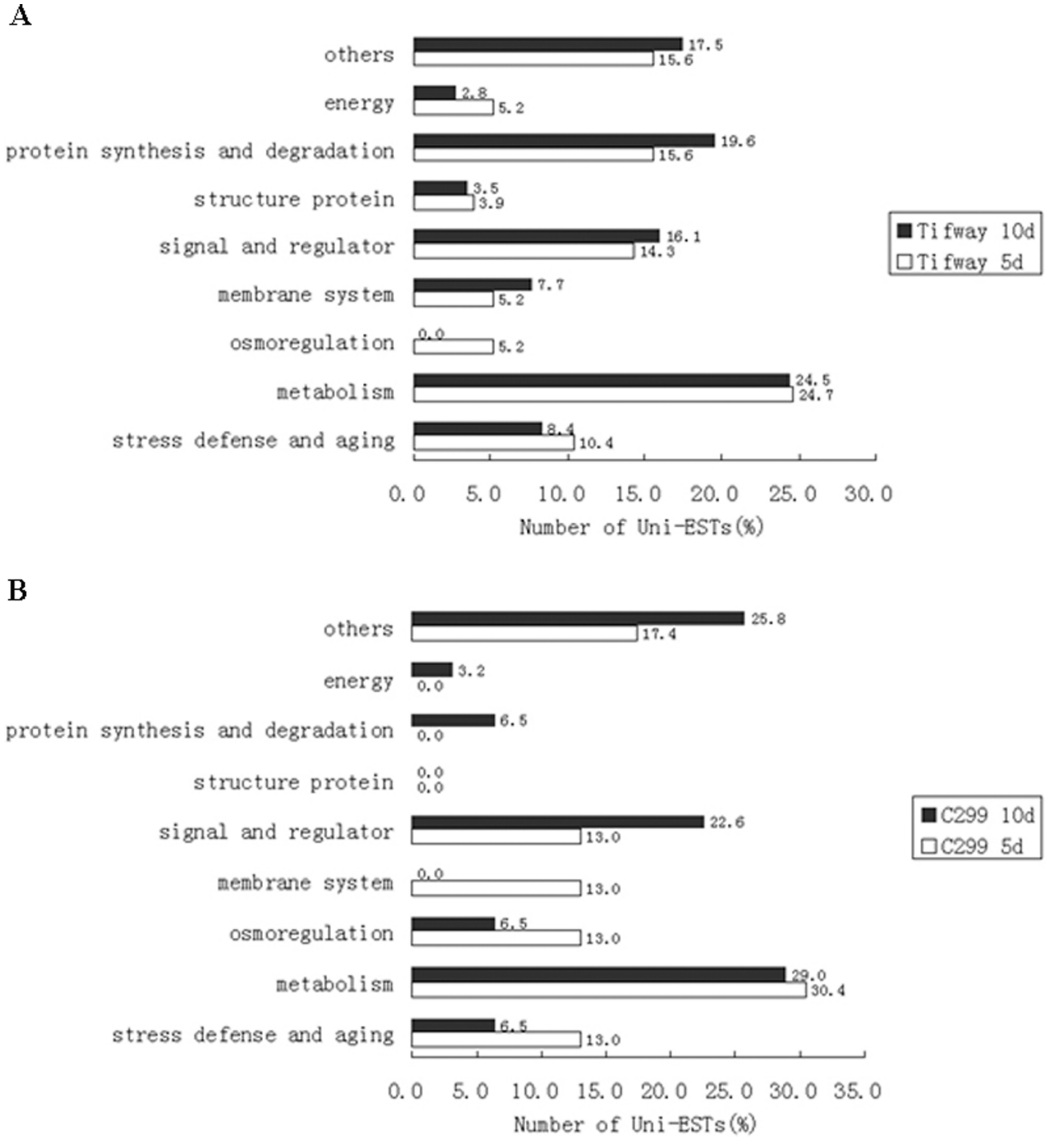
Comparison of the percentages of gene transcripts in each functional classification between 5-d drought and 10-d drought SSH libraries of ‘Tifway’ and ‘C299’. A: Comparison for ‘Tifway’; B: Comparison for ‘C299’. The bars represent the percentages of gene transcripts for both genotypes in each functional category.

The percentages of drought-responsive genes were compared between the two genotypes. Most of the up-regulated genes in ‘C299’ with known functions were involved in metabolism (30.4% at 5-d drought, 29.0% at 10-d drought), stress defense (13.0% at 5-d drought, 6.5% at 10-d drought), and signal and regulator (13.0% at 5-d drought, 22.6% at 10-d drought) (Fig. 3A). For ‘Tifway’, most of the up-regulated genes were classified in metabolism (24.7% at 5-d drought, 24.5% at 10-d drought), stress defense (10.4% in drought 5-d, 8.4% at 10-d drought), and signal and regulators (14.3% at 5-d drought, 16.1% at 10-d drought) (Fig. 2B). In addition, a large proportion of up-regulated genes in the protein synthesis and degradation category (15.6% at 5-d drought, 19.6% at 10-d drought) were detected in ‘Tifway’, but a small fraction of those genes were detected in ‘C299’ (6.5% at 10-d drought, 0% at 5-d drought) (Fig. 3B).

The percentages of up-regulated gene transcripts in both genotypes changed with drought stress duration or degree of severity. A higher percentage of up-regulated genes were found in the category protein synthesis and degradation in both ‘Tifway’ and ‘C299’ plants exposed to 10-d drought than those at 5-d drought. However, the percentage of genes in osmoregulation was higher in both ‘Tifway’ and ‘C299’ plants exposed to 5-d drought than those at 10-d drought. The percentage of up-regulated genes in membrane system was also higher in ‘C299’ exposed to 5-d drought than those at 10-d drought, while for ‘Tifway’, the percentage of genes in membrane system was lower at 5 d of drought than those at 10 d of drought.

### Q-PCR confirmation and comparative analysis of transcript levels for drought up-regulated genes between ‘C299’ and ‘Tifway’

Thirty seven genes up-regulated by drought in either or both genotypes and differentially expressed at different transcript levels between the two genotypes at 5 and 10 d of drought that were identified from the SSH analysis were further examined using Real time-PCR. The Real time-PCR analysis confirmed that 36 genes displayed differential expression levels between the two bermudagrass genotypes under drought conditions (Figure S1). One gene, CdL10–39 (JK340529, putative starch branching enzyme 4), did not exhibit differences in its expression level between the two genotypes at 0, 5, or 10 d of drought.

**Figure 4:**
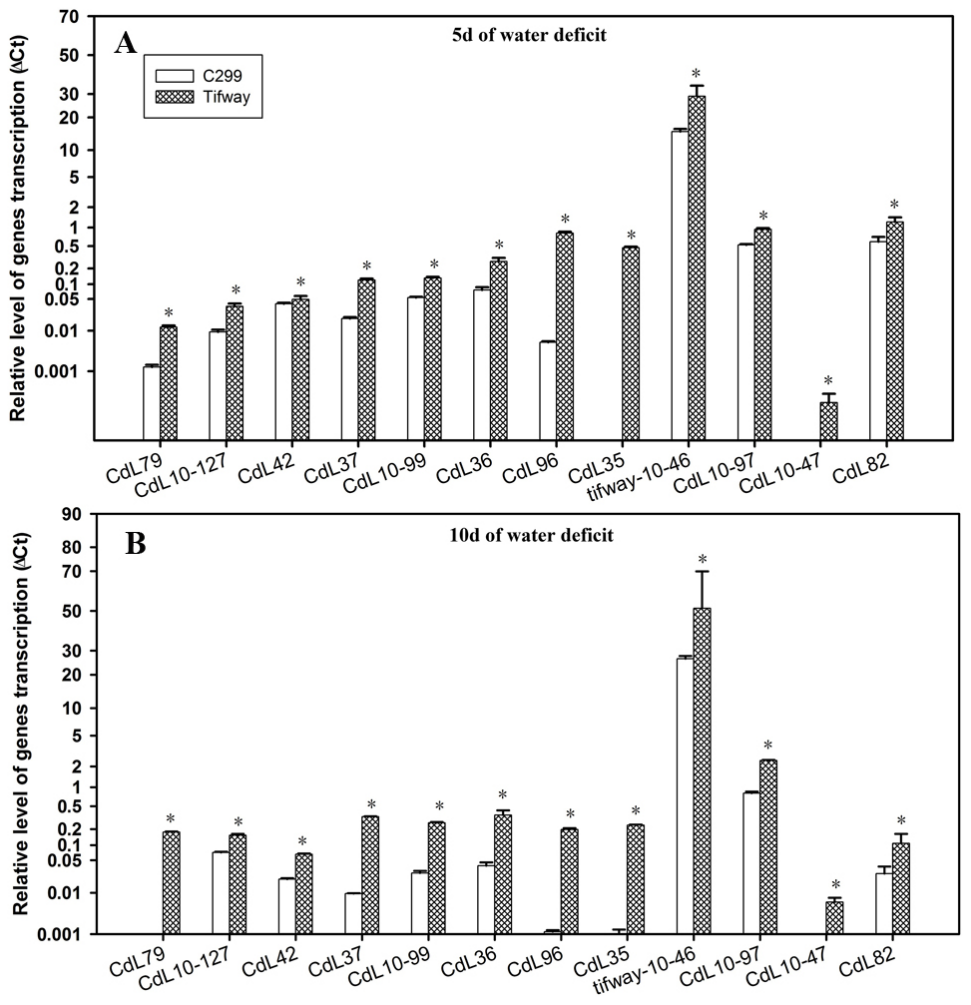
Q-PCR analysis of expression of 12 genes from four SSH libraries, exhibiting higher levels in ‘Tifway’ and ‘C299’ at 5 and 10 d of drought. A; Comparison of the relative gene expression levels of ‘Tifway’ and ‘C299’ at 5 d of drought; B: Comparison of the relative gene expression levels of ‘Tifway’ and ‘C299’ at 10 d of drought. The error bars represent standard deviations of the means. For each gene, the significant differences based on LSD test at P = 0.05 between ‘Tifway’ and ‘C299’ were shown by asterisk on the top of the error bars.

Twelve genes had significantly higher levels of expression in ‘Tifway’ than ‘C299’ at 5 and 10 d of drought (Fig. 4). These genes included CdL79 (JK340618, putative WRKY transcription factor 50), CdL10–127 (JK340747, putative DHAR), CdL42 (JK340615, putative EREBP-4 like protein), CdL37 (JK340586, putative cytosolic NADP malic enzyme), CdL10–99 (JK340714, putative 2-cys peroxiredoxin), CdL36 (JK340585, putative sterol desaturase family protein), CdL96 (JK340598, putative CER1), CdL35 (JK340626, putative ATP-dependent Clp protease ATP-binding subunit precursor), Tifway-10–46 (JK340856, putative dehydrin(DHN4)), CdL10–97 (JK340737, putative GAPDH - glyceraldehyde-3-phosphate dehydrogenase), CdL10–47 (JK340720, putative aldehyde oxidase), and CdL82 (JK340582, putative HVA22-like protein).

**Figure 5:**
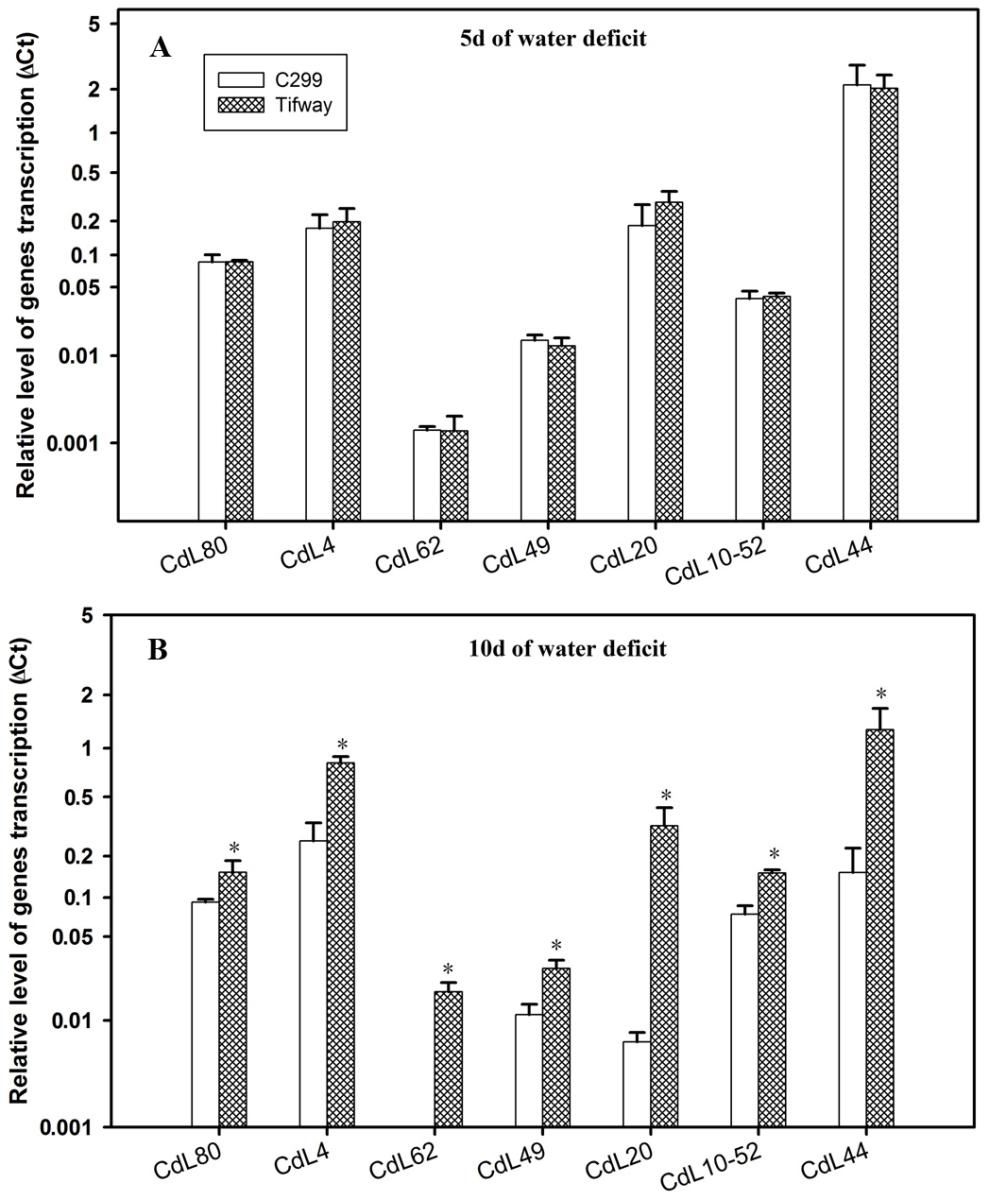
Q-PCR analysis of expression levels of seven genes from four SSH libraries, exhibiting higher expression in ‘Tifway’ than in ‘C299’ only 10 d of drought. Comparison of the relative gene expression levels of ‘Tifway’ and ‘C299’ at 5 d of drought; B: Comparison of the relative gene expression levels of ‘Tifway’ and ‘C299’ at 10 d of drought. The error bars represent standard deviations of the means. For each gene, the significant differences based on LSD test at P = 0.05 between ‘Tifway’ and ‘C299’ were shown by asterisk on the top of the error bars.

Seven genes had significantly higher level of expression in ‘Tifway’ than in‘C299’ at 10 d of drought, but no significant differences were detected between two genotypes at 5 of drought (Fig. 5). The genes were CdL80 (JK340610, putative Remorin), CdL4 (JK340476, putative DNA repair ATPase-related), CdL62 (JK340580, putative leaf senescence related protein-like), CdL49 (JK340642, putative LEC14B protein), CdL20 (JK340484, putative permease I), CdL10–52 (JK340708, putative superoxide dismutase [Cu-Zn]), and CdL44 (JK340578, putative SK3-type dehydrin).

**Figure 6:**
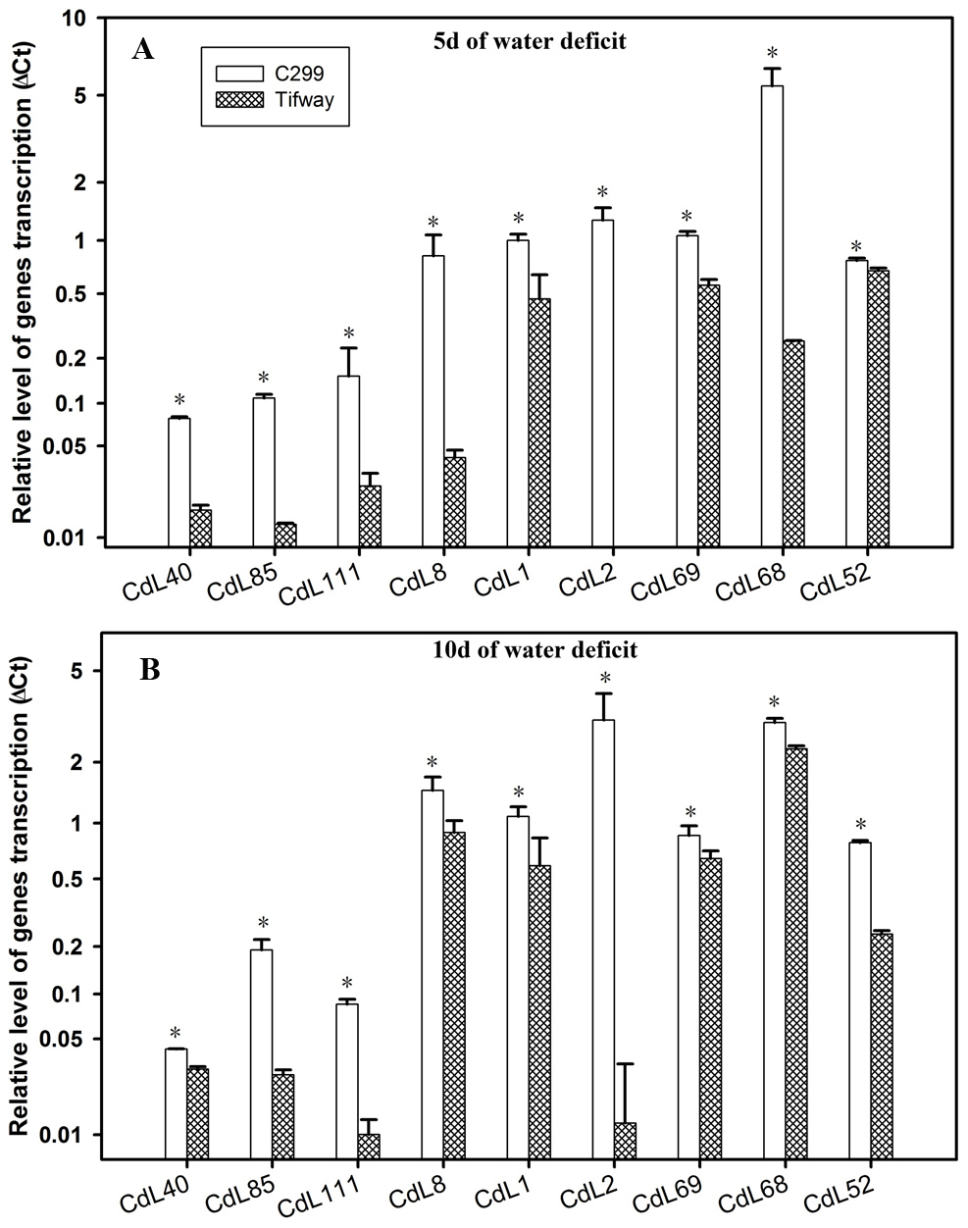
Q-PCR analysis of expression of nine genes from four SSH libraries, exhibiting higher levels in ‘C299’ than in ‘Tifway’ at 5 and 10 d of drought. Comparison of the relative gene expression levels of ‘Tifway’ and ‘C299’ at 5 d of drought; B: Comparison of the relative gene expression levels of ‘Tifway’ and ‘C299’ at 10 d of drought. The error bars represent standard deviations of the means. For each gene, the significant differences based on LSD test at P = 0.05 between ‘Tifway’ and ‘C299’ were shown by asterisk on the top of the error bars.

Nine genes showed significantly lower expression levels in ‘Tifway’ than in ‘C299’ at both 5 and 10 d of drought (Fig. 6). These genes were CdL40 (JK340589, putative 4-hydroxyphenylpyruvate dioxygenase), CdL85 (JK340595, putative pyruvate dehydrogenase E1 alpha subunit), CdL111 (JK340584, maturation-associated protein), CdL8(JK340518, putative PR17c precursor), CdL1 (JK340473, putative Cytokine induced apoptosis inhibitor), CdL2(JK340477, putative alcohol dehydrogenase), CdL69 (JK340599, putative delta 1-pyrroline-5-carboxylate synthetase), CdL68 (JK340608, putative stachyose synthase), and CdL52(JK340628, putative protein phosphatase 2C).

**Figure 7:**
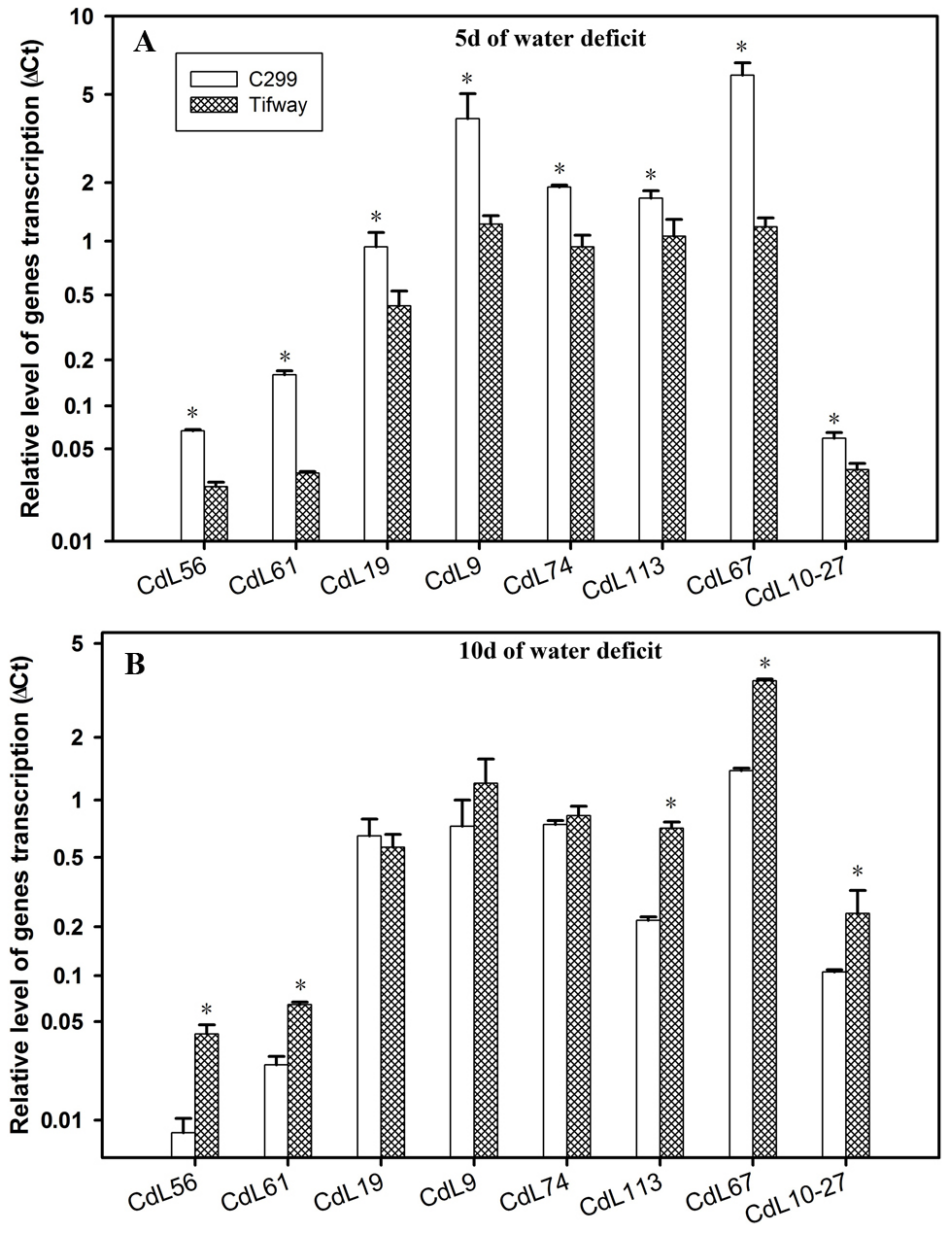
Q-PCR analysis of expression of eight genes from four SSH libraries, exhibiting higher levels in ‘C299’ than in ‘Tifway’ only at 5 d of drought. Comparison of the relative gene expression levels of ‘Tifway’ and ‘C299’ at 5 d of drought; B: Comparison of the relative gene expression levels of ‘Tifway’ and ‘C299’ at 10 d of drought. The error bars represent standard deviations of the means. For each gene, the significant differences based on LSD test at P = 0.05 between ‘Tifway’ and ‘C299’ were shown by asterisk on the top of the error bars.

Eight genes exhibited significantly lower levels of expression in ‘Tifway’ than in ‘C299’ only at 5 d of drought (Fig. 7A).They included CdL56(JK340591, putative beta-galactosidase), CdL61 (JK340592, gibberellin 20 oxidase 2), CdL19 (JK340483, putative sucrose synthase), CdL9 (JK340478, putative carbonic anhydrase), CdL74 (JK340625, putative actin depolymerization factor-like protein), CdL113 (JK340639, putative CTP synthase), CdL67 (JK340607, putative galactinol synthase 1), CdL10–27 (JK340525, putative plastid starch synthase I precursor). At 10 d of drought, CdL56, CdL61, CdL67, CdL113, and CdL10–27 had significantly higher expression level in ‘Tifway’ than ‘C299’ and other three did not exhibit genotypic differences (Fig. 7B).

### Hierarchical clustering analysis of up-regulated genes under drought

**Figure 8:**
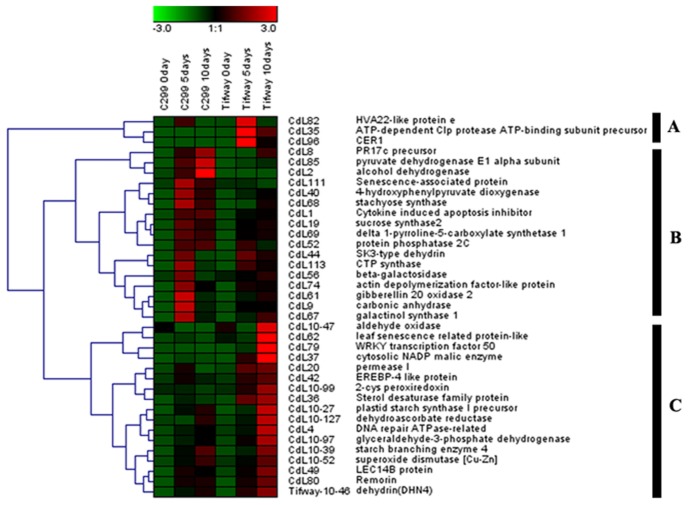
Cluster analysis of 37 genes differentially-expressed between well-watered control and drought in ‘Tifway’ and ‘C299’. Cluster analysis for each group of genes was performed using hierarchical clustering of Genesis 1.7.5 with average linkage and Euclidian distance measurement. Rows represent differentially-expressed genes, while columns represent the genotypes with 5 d or 10 d of drought treatment. Each gene expressed in different conditions is presented as a cell. Red, green, and black boxes represent genes that increased, decreased, and had equal expression levels at a given duration of drought treatment, respectively. The gene name and annotation of each gene are listed on the right of the figure, and the cluster numbers are listed on the left. The color scale shown at the top illustrates the relative expression ratios of gene across all samples.

Hierarchical clustering analysis was performed to analyze the similarity of the expression patterns for 37 up-regulated genes based on average linkage and Euclidean distance as a measurement of similarity by using Genesis, which provides information on co-expressed genes [23]. The 37 up-regulated genes by drought in either or both genotypes were clustered into three groups with similar expression patterns for genes within each group (Fig. 8).

Group A included three genes, CdL82 (JK340582, putative HVA22-like protein, CdL96 (JK340598, putative CER1), and CdL35 (JK340626, putative ATP-dependent Clp protease ATP-binding subunit precursor). The expression level of genes in Group A increased at 5 d of drought, to a greater extent in ‘Tifway’ than in ‘C299’, but decreased as drought was prolonged to 10 d in both genotypes.

Group B had 17 genes, including five genes involved in osmotic regulation - CdL19 (JK340483, putative sucrose synthase), CdL68 (JK340608, putative stachyose synthase), CdL56 (JK340591, putative beta-galactosidase), and CdL67 (JK340607, putative galactinol synthase 1), and CdL69 (JK340599, putative delta 1-pyrroline-5-carboxylate synthetase 1). In Group B, the expression level of most genes except CdL8 (JK340518, putative PR17c precursor), CdL2 (JK340477, putative alcohol dehydrogenase), and CdL85 (JK340595, putative pyruvate dehydrogenase E1 alpha subunit) decreased to a lower level at 10 d of drought compared to that at 5 d in ‘C299’, but the gene expression level was significantly higher in ‘C299’ than in ‘Tifway’ at 5 and 10 d of drought.

Group C had 17 genes, including four genes associated with ROS scavenging - CdL10–52 (JK340708, putative superoxide dismutase [Cu-Zn]), CdL10–99 (JK340714, putative 2-Cys peroxiredoxins), CdL10–47 (JK340720, putative aldehyde oxidase), and CdL10–127(JK340747, putative dehydroascorbate reductase). Group C also included CdL10–97 (JK340737, putative GAPDH) with diverse functions and a gene associated with dehydration protection (Tifway-10–46, JK340856, putative dehydrin (DHN4). Genes in Group C exhibited increased level of expression with drought at both 5 and 10 d, and had significantly higher expression level in ‘Tifway’ than ‘C299’.

## Discussion

Genotypic variations in differential gene expression in response to drought stress are also reflected at the physiological levels. Physiological analysis with ‘Tifway’ and ‘C299’ exposed to drought stress demonstrated that ‘Tifway’ was able to maintain higher cell membrane stability and water status (Table 2), as well as greater photosynthetic rate, photochemical efficiency, and antioxidant defenses [16]–[18]. The physiological data suggested that ‘Tifway’ exhibited superior drought resistance to ‘C299’. The gene expression analysis in this study provided further insights on molecular factors associated with superior drought resistance in ‘Tifway’ bermudagrass, as manifested by the physiological traits.

Drought avoidance is a key strategy in drought resistance of plants, which ischaracterized by minimizing water loss through various mechanisms such as formation of wax on leaf surfaces, which provides primary protection against leaf dehydration (Post-Beittenmiller, 1996; Islam et al., 2009) [24], [25]. A CER1 gene (CdL96, JK340598) which encodes a protein involved in a key step in wax biosynthesis [26] was detected at 5 and 10 d of drought stress, with the expression level being higher in drought-tolerant ‘Tifway’ than in drought-sensitive ‘C299’. Another gene, sterol desaturase family protein (CdL36, JK340585), putatively encoding a sterol desaturase that has been reported to be involved in epicuticular wax biosynthesis [25], was also much more highly expressed in ‘Tifway’ than in ‘C299’ at 5 and 10 d of drought stress. In a previous study, water loss rate from leaves was significantly slower in ‘Tifway’ than ‘C299’ under drought stress [17], which was corresponded to the enhanced expression of genes controlling wax synthesis in ‘Tifway’. The up-regulation of CER1 and sterol desaturase may enhance cuticle wax synthesis, allowing plants to avoid dehydration. ‘Tifway’ indeed exhibited higher RWC in leaves compared to ‘C299’ under drought stress.

Drought tolerance mechanisms have been associated with cellular protection from dehydration by accumulating protective proteins, most notably dehydrins and other late-embryogenesis abundant (LEA) proteins [27]. Although the function of many dehydrins and LEA proteins is not fully understood, at least part of their function is to act as chaperones that protect protein and membrane structure [28], [29]. Compatible solutes can also protect protein and membrane structure under dehydration [30]. In ‘Tifway’ plants exposed to 10 d of drought, one EST (Tifway-10–46, JK340856) showed high homology to DHN4 gene [*Hordeum vulgare*] with E-value 2.00E-98, which is putative encoding dehydrin protein. Q-PCR result confirmed that its expression level increased with progressive drought and was significantly higher in ‘Tifway’ than ‘C299’. The expression pattern of DHN4 gene in the current study was similar to the expression of 40-kDa dehydrins in the report by Hu et al. [17] for the same cultivar ‘Tifway’ exposed to 10 d of drought stress. Hu et al. [17] detected multiple dehydrin expression patterns in ‘Tifway’ and ‘C299’ under drought stress by immunoblotting analysis, and the level of expression of 40-kDa dehydrin was positively associated with drought tolerance in bermudagrass. Our results at the transcript level in combination with that at the protein level [17] suggested that dehydrins may play an important role in protecting bermudagrass leaves from dehydration. Another gene, *HAV22* (CdL82, JK340582), which was reported to be involved in ABA-induced gene expression and have some homolog with LEA protein [31], was expressed significantly higher in ‘Tifway’ than in ‘C299’ at 5 and 10 d of drought stress. A GC-rich element from barley HVA22 gene was reported to be essential for ABA or stress-inducible gene expression [32]. But the expression of *HAV22* decreased significantly at 10 d of stress in ‘Tifway’, revealing *HAV22* expression may have short-term effects on stress defense.

Another aspect of drought tolerance is the control of the level of ROS or minimizing the damage caused by ROS. In our experiments, some genes involved in ROSs scavenging were detected in ‘Tifway’ plants exposed to 10 d of drought stress, including superoxide dismutase (SOD; CdL10–52, JK340708), aldehyde oxidase (CdL10–47, JK340720), dehydroascorbate reductase (DHAR; CdL10–127, JK340747), and 2-cys peroxiredoxin bas1 (CdL10–99, JK340714). SOD catalyzes the dismutation of superoxide into oxygen and hydrogen peroxide [33]. Aldehyde oxidase (AO) produces hydrogen peroxide and under certain conditions can catalyze the formation of superoxide [34]. DHAR is responsible for regenerating ascorbic acid (Asc) from an oxidized state, regulates the cellular Asc redox state, which in turn affects cell responsiveness and tolerance to environmental ROS [35]. Plant 2-Cys peroxiredoxins are post-translationally targeted to chloroplasts, protecting the photosynthetic membrane against photooxidative damage [36]. Q-PCR analysis confirmed that these genes had increased expression level in the drought-tolerant genotype. Under drought stress, their expression level was significantly higher in ‘Tifway’ than in ‘C299’, especially at 10 d. Zhao et al. [18] also reported high abundance of SOD protein in ‘Tifway’ than in ‘C299’ at 10 d of drought stress. Chen et al. [37] found bermdagrass mutants with superior drought tolerance had than the wild type. The results at both transcript and protein levels indicated that the maintenance of active antioxidant genes and proteins could contribute to the superior drought tolerance in ‘Tifway’, as manifested by greater cell membrane stability (Table 2) The hierarchical clustering analysis revealed that glyceraldehyde-3-phosphate dehydrogenase (GAPDH) (CdL10–97, JK340737) was closely related to the four genes involved in ROSs scavenging. By correlation coefficient analysis, the expression of CdL10–97 had high positive correlation coefficient with CdL10–52 and CdL10–127 which were related to ROSs scavenging (r>0.8, p<0.05), revealing GAPDH may had other functions related to ROSs scavenging. GAPDH is an enzyme that catalyzes the sixth step of glycolysis. But increasing evidence suggests that GAPDH is multifunctional, it displays a number of diverse activities unrelated to its glycolytic function [38] such as a signaling role in mediating ROS responses [39]–[42].

Osmotic adjustment or regulation has also been considered an important drought tolerance mechanism, which is accomplished through the accumulation of solutes in plants, contributing to cell turgor maintenance during drought [43]. Genes classified in the osmoregulation category were detected at 5 d of drought stress in both genotypes, but the percentage of genes in this category decreased with stress duration up to 10 d in both genotypes. These data suggested that osmotic regulation could be involved in early response of bermudagrass to drought stress, but not long-term drought tolerance in this species. Drought up-regulated genes with putative functions as solute synthase were detected in both genotypes of bermudagrass exposed to drought stress, including sucrose synthase2 (CdL19, JK340483), galactinol synthase 1(CdL67, JK340607), stachyose synthase (CdL68, JK340608) and delta 1-pyrroline-5-carboxylate synthetase 1(CdL69, JK340599), but the expression level was lower in the drought-tolerant than in the drought-sensitive genotype. Sucrose synthase, galactinol synthase, and stachyose synthase are related to the synthesis of oligosaccharides, which are involved in osmotic adjustment [44]. Delta 1-pyrroline-5-carboxylate synthetase catalyzes proline synthesis [45]. Previous studies have shown that proline accumulate was responsive to drought stress and serves as a protective solute to maintain cell turgor against dehydration in various plant species [46], oxidative protection [47], and function as molecular chaperone stabilizing the structure of proteins [48]. The up-regulation of those genes associated with solute accumulation under drought stress, particularly in the drought-sensitive genotype reflected that sugar and proline accumulation was sensitive to mild or short-term drought stress in bermudagrass, but may not contribute to superior drought tolerance in this species under long-term stress.

Several genes involved in other functional categories were also found differentially expressed between the two genotypes under drought conditions. CdL35 (JK340626) was confirmed up-regulated in ’Tifway’ at 5 and 10 d of drought stress, and presented a higher expression level in ‘Tifway’ than in ‘C299’. This gene is encoding a subunit of ATP-dependent Clp protease. ATP-dependent Clp protease is involved in a variety of processes, ranging from developmental changes to stress tolerance [49], [50]. CdL37 (JK340586) is encoding cytosolic NADP malic enzyme, were expressed at a significantly higher level in ‘Tifway’ than in ‘C299’ at both 5 and 10 d of drought stress. Cytosolic NADP malic enzyme is related to plant defense to various abiotic stresses [51]. Other genes that were differentially expressed between ‘C299’ and ‘Tifway’ included transcription factors and genes regulated by those transcription factors. Transcription Factors (TFs) EREBP-4 like protein (CdL42, JK340615) and WRKY transcription factor 50 (CdL79, JK340618) were expressed at a significantly higher level in ‘Tifway’ than in ‘C299’ at 5 and 10 d of drought. EREBP-4 like protein (CdL42, JK340615) belongs to AP2/EREBP family members, which bind DRE “TACCGACAT” motifs and regulate a series of drought responsive genes [52]. Many genes activated by AP2/EREBP TFs, such as *rd29A*, were reported to be associated with better drought tolerance [53], [54]. The WRKY genes encode a large group of transcription factors. They bind specifically to W box motif “(T)(T)TGAC(C/T)” which was presented in many co-regulated defense gene promoters [55]. Over-expression of OsWRKY11 in rice (*Oryza sativa*) enhanced drought tolerance [56]. The differential expression of these transcription factors between the two genotypes suggest that the downstream responses to drought stress varied with genotypes differing in drought tolerance. Leaf senescence related protein-like (CdL62, JK340580) regulated by WRKY gene was more highly expressed in ‘Tifway’ than ‘C299’. It may take part in initiating the process of leaf senescence induced by drought [57], [58], which has been associated with plant survival of drought stress by reducing leaf area for transpiration to limit water loss from the plant canopy and diverting carbon partitioning.

### Summary

The SSH analysis identified 277 drought-responsive genes in two genotypes of bermudagrass in response to mild or short-term drought (5 d) and severe or long-term (10 d) drought stress. Those genes were mainly classified in the functional categories of stress defense and aging, metabolism, osmoregulation, membrane system, signal and regulator, structure protein, protein synthesis and degradation, and energy metabolism. Q-PCR analysis confirmed the expression of 36 drought up-regulated genes that were more highly expressed in drought-tolerant ‘Tifway’ than drought-sensitive ‘C299’ at 5 and 10 d of drought stress, such as those for leaf cuticular wax accumulation (CER1, sterol desaturase), stress signaling (EREBP-4 like protein, WRKY transcription factor), dehydration protective proteins (dehydrins and HVA22-like protein), and oxidative stress defense (SOD, DHAR, 2-Cys peroxiredoxins, GAPDH) for bermudagrass tolerance to drought. Molecular markers for the above-mentioned genes could be utilized for marker-assisted selection of drought-tolerant warm-season perennial grasses. This approach is particularly relevant for grasses grown in shallow soil profiles.

## Supporting Information

Figure S1 **Q-PCR analysis of all the 37 genes expression in both genotypes under 0-d, 5-d, and 10-d water deficit.** For each gene, the significant differences based on LSD test at P = 0.05 between ‘Tifway’ and ‘C299’ were shown by different letters on the top of the error bars.
(TIF)

File S1 **Supporting tables from Table S1 to Table S8 of four SSH libraries clones analyzed by BLAST.**
(DOC)
