# Supplementary figures and images for: Characterization of Gene Expression Associated with Drought Avoidance and Tolerance Traits in a Perennial Grass Species
Source: PLoS One. 2014 Aug 25;9(8):e103611. doi: 10.1371/journal.pone.0103611 (PMC4143173; doi:10.1371/journal.pone.0103611)

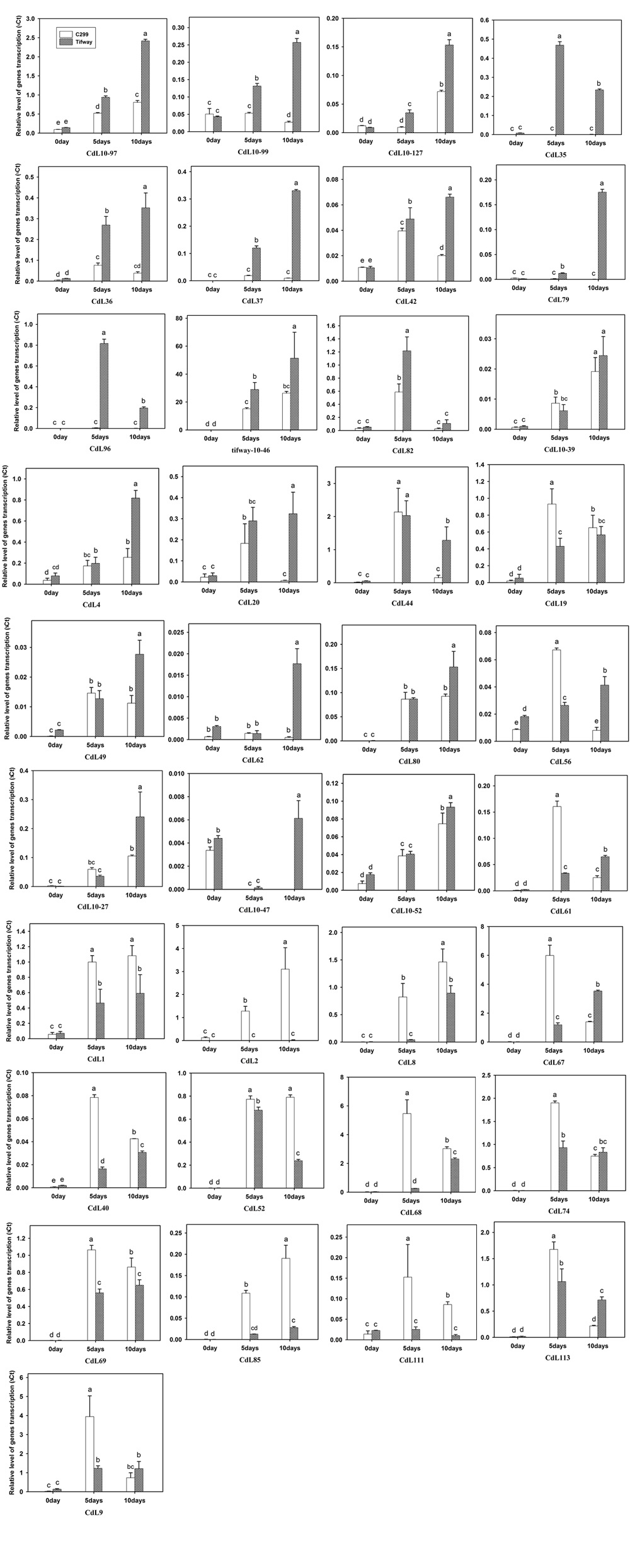

Supplement: Figure S1 — Q-PCR analysis of all the 37 genes expression in both genotypes under 0-d, 5-d, and 10-d water deficit. For each gene, the significant differences based on LSD test at P = 0.05 between ‘Tifway’ and ‘C299’ were shown by different letters on the top of the error bars. (TIF) [file pone.0103611.s001.tif]
