# Supplementary material for: Characterization of Gene Expression Associated with Drought Avoidance and Tolerance Traits in a Perennial Grass Species
Source: PLoS One. 2014 Aug 25;9(8):e103611. doi: 10.1371/journal.pone.0103611 (PMC4143173; doi:10.1371/journal.pone.0103611)
Supplement: File S1 — Supporting tables from Table S1 to Table S8 of four SSH libraries clones analyzed by BLAST. (DOC) [file pone.0103611.s002.doc]

**Supplementary data files:**

Table S1 BlastX and BLastN analysis of ‘C299’ drought 5 days SSH library clones

| Name | Accession  number | Length  (bp) | Gene identification  (Similar gene in other speices) | Similar gene accession  number | E-value |
| --- | --- | --- | --- | --- | --- |
|  |  |  | **stress 、 defense and aging** |  |  |
| CdL5 | JK340474 | 230 | heat shock protein binding [Arabidopsis thaliana] | NP_181633.3 | 1.00E-07 |
| CdL18 | JK340475 | 164 | salt-responsive WD40 protein 5 [Oryza sativa Japonica Group] | ACD99699.1 | 2.00E-23 |
| CdL8 | JK340518 | 273 | PR17c precursor(PR17 family)[ Hordeum vulgare subsp. vulgare] | ABV22582.1 | 2.00E-21 |
|  |  |  |  |  |  |
|  |  |  | **metabolism** |  |  |
| CdL2 | JK340477 | 309 | alcohol dehydrogenase 1 [Miscanthus sinensis var. formosanus] | CAD56719.1 | 1.00E-51 |
| CdL9 | JK340478 | 315 | carbonic anhydrase [Zea mays] | NP_001149686.1 | 3.00E-18 |
| CdL24 | JK340479 | 218 | 'slow' alcohol dehydrogenas mRNA[Phragmites australis] | AY917130.2 | 0.072 |
| CdL6 | JK340480 | 266 | glutaryl-CoA dehydrogenase [Zea mays] | NP_001148492.1 | 7.00E-54 |
| CdL27 | JK340481 | 383 | serine decarboxylase [Brassica napus] | BAA78331.1 | 8.00E-71 |
| CdL7 | JK340482 | 165 | Aspartate/tyrosine/aromatic aminotransferase [Micromonas sp.RCC299] | XP_002509287.1 | 0.009 |
| CdL4 | JK340476 | 205 | DNA repair ATPase-related [Arabidopsis thaliana] | NP_565569.1 | 8.00E-021 |
|  |  |  |  |  |  |
|  |  |  | **osmoregulation** |  |  |
| CdL19 | JK340483 | 247 | sucrose synthase2 [Zea mays] | NP_001105194.1 | 5.00E-41 |
| CdL20 | JK340484 | 220 | permease I [Zea mays] | ACG35525.1 | 4.00E-35 |
| CdL15 | JK340485 | 291 | putative insulin degrading enzyme [Oryza sativa Japonica Group] | BAD52841.1 | 8.00E-50 |
|  |  |  |  |  |  |
|  |  |  | **membrane system** |  |  |
| CdL12 | JK340486 | 265 | putative mitochondrial carrier protein [Oryza sativa Japonica Group] | AAV43843.1 | 1.00E-36 |
| CdL13 | JK340487 | 343 | vesicle-associated membrane protein 725 [Zea mays] | NP_001148792.1 | 5.00E-71 |
| CdL17 | JK340488 | 504 | membrane protein-like protein [Glycine max] | ABC47859.1 | 6.00E-16 |
|  |  |  |  |  |  |
|  |  |  | **regulation** |  |  |
| CdL14 | JK340489 | 279 | casein protein kinase 2 alpha subunit [Lolium perenne] | BAD98470.1 | 2.00E-34 |
| CdL16 | JK340490 | 427 | putative cell division cycle protein 48 [Oryza sativa (japonica cultivar-group)] | AAP53974.2 | 2.00E-91 |
| CdL1 | JK340473 | 456 | Cytokine induced apoptosis inhibitor[Arabidopsis thaliana] | NP_598902.1 | 8.00E-05 |
|  |  |  |  |  |  |
|  |  |  | **others** |  |  |
| CdL10 | JK340491 | 358 | DEAD/DEAH box helicase family protein [Oryza brachyantha] | ABG73436.1 | 2.00E-61 |
| CdL21 | JK340492 | 225 | hypothetical protein mRNA [Zea mays] | EU963487.1 | 1.00E-05 |
| CdL22 | JK340493 | 93 | hypothetical protein mRNA SORBIDRAFT_04g037650 [Sorghum bicolor] | XM_002453016.1 | 1.00E-11 |
| CdL25 | JK340494 | 198 | hypothetical protein mRNA [Sorghum bicolor] | XM_002465529.1 | 2.00E-34 |
|  |  |  |  |  |  |

| Name  Table S2 BlastN (with the EST others database) analysis of ‘C299’ drought 5 days SSH library clones which had no match with reported genes or proteins | Accession  number | Length  (bp) | Gene identification | Similar gene accession  number | E-value |
| --- | --- | --- | --- | --- | --- |
| C299-5-1 | JK340495 | 77 | no significant homology |  |  |
| C299-5-4 | JK340496 | 95 | dehydrin-/LEA group 2-like protein mRNA [Cleistogenes songorica] | FJ972827.1 | 2.00E-4 |
| C299-5-5 | JK340497 | 96 | Bermudagrass cDNA [Cynodon dactylon ] | ES297016.1 | 3.00E-25 |
| C299-5-7 | JK340498 | 98 | Bermudagrass Normalized cDNA [Cynodon dactylon ] | ES301958.1 | 2.00E-40 |
| C299-5-8 | JK340499 | 127 | no significant homology |  |  |
| C299-5-10 | JK340500 | 116 | roots (15 days 300mM NaCl) cDNA [Aeluropus littoralis ] | EE594556.1 | 2.00E-11 |
| C299-5-11 | JK340501 | 117 | Bermudagrass cDNA similar to H-transporting ATP synthase[Cynodon dactylon ] | BG322274.1 | 3.00E-52 |
| C299-5-12 | JK340502 | 123 | Panicum virgatum callus (N)[Panicum virgatum ] | GD002802.1 | 1.00E-13 |
| C299-5-15 | JK340503 | 139 | MA_LYP9_07314 Mature embryo cDNA Library of LYP9 Oryza sativa  Indica Group cDNA 5', mRNA sequence[Oryza sativa (Indica -group)] | FG968530.1 | 3.00E-9 |
| C299-5-16 | JK340504 | 142 | no significant homology |  |  |
| C299-5-18 | JK340505 | 151 | (Kaye Murri) root Eragrostis tef cDNA [root7_m5.y1.abd] | DN483538.1 | 2.00E-24 |
| C299-5-19 | JK340506 | 155 | no significant homology |  |  |
| C299-5-20 | JK340507 | 159 | no significant homology |  |  |
| C299-5-22 | JK340508 | 162 | root salinity induced expressed (EST)[ Spartina alterniflora] | EH277771.1 | 4.00E-34 |
| C299-5-24 | JK340509 | 171 | Bermudagrass Normalized cDNA[Cynodon dactylon ] | ES296812.1 | 2.00E-26 |
| C299-5-25 | JK340510 | 179 | Cleistogenes songorica drought stress induced library cDNA, mRNA sequence.[Cleistogenes songorica] | JG393813.1 | 1.00E-10 |
| C299-5-27 | JK340511 | 189 | roots (15 days 300mM NaCl) cDNA [Aeluropus littoralis ] | EE594547.1 | 1.00E-47 |
| C299-5-28 | JK340512 | 191 | Bermudagrass cold acclimated cDNA [Cynodon dactylon ] | DN985603.1 | 2.00E-89 |
| C299-5-32 | JK340513 | 222 | Bermudagrass Normalized cDNA [Cynodon dactylon ] | ES293077.1 | 3.00E-82 |
| C299-5-35 | JK340514 | 238 | no significant homology |  |  |
| C299-5-38 | JK340515 | 148 | Panicum virgatum callus (N) cDNA [Panicum virgatum] | FL986296.1 | 1.00E-28 |
| C299-5-39 | JK340516 | 304 | Bermudagrass cold acclimated cDNA [Cynodon dactylon ] | DN987646.1 | 3.00E-153 |
| C299-5-41 | JK340517 | 329 | Bermudagrass Normalized cDNA [Cynodon dactylon ] | ES292116.1 | 2.00E-130 |

| Name  Table S3 BlastX and BLastN analysis of ‘Tifway’ drought 5 days SSH library clones | Accession  number | Length  (bp) | Gene identification | Similar gene accession  number | E-value |
| --- | --- | --- | --- | --- | --- |
|  |  |  | **stress, defense and aging** |  |  |
| CdL43 | JK340577 | 163 | salt-stress induced hydrophobic peptide (ESI3) gene [Lophopyrum elongatum] | U00966 | 2.00E-20 |
| CdL44 | JK340578 | 94 | SK3-type dehydrin mRNA [Oryza sativa (Japonica Group)] | EF444534.1 | 2.00E-21 |
| CdL54 | JK340579 | 405 | peroxisomal targeting signal 1 receptor short or long form [Oryza sativa (japonica cultivar-group)] | AAX11256.1 | 1.00E-25 |
| CdL62 | JK340580 | 296 | leaf senescence related protein-like [Oryza sativa Japonica Group] | BAD81676.1 | 2.00E-54 |
| CdL81 | JK340581 | 31 | heat shock 70kDa protein 6 (HSP70B') [Bos taurus] | XM_002685850.1 | 0.005 |
| CdL82 | JK340582 | 140 | HVA22-like protein e [Oryza sativa Japonica Group] | ABA93823.1 | 7.00E-25 |
| CdL83 | JK340583 | 227 | heavy metal-associated domain containing protein[Zea mays] | NM_001157058.1 | 1.00E-06 |
| CdL111 | JK340584 | 619 | Senescence-associated protein, expressed [Oryza sativa (japonica cultivar-group)] | ABF94900.1 | 6.00E-71 |
|  |  |  |  |  |  |
|  |  |  | **metabolism** |  |  |
| CdL36 | JK340585 | 226 | Sterol desaturase family protein, expressed [Oryza sativa (japonica cultivar-group)] | ABF93565.1 | 2.00E-33 |
| CdL37 | JK340586 | 88 | cytosolic NADP malic enzyme [Oryza sativa (indica cultivar-group)] | AAR15892.1 | 8.00E-10 |
| CdL38 | JK340587 | 93 | Zea mays clone 244285 diphosphomevalonate decarboxylase mRNA [Zea mays] | EU962624.1 | 9.00E-08 |
| CdL40 | JK340589 | 169 | 4-hydroxyphenylpyruvate dioxygenase [Triticum aestivum] | AAZ67144.1 | 7.00E-28 |
| CdL53 | JK340590 | 40 | 1-aminocyclopropane-1-carboxylate oxidase mRNA [Oryza sativa] | X85747.1 | 0.01 |
| CdL56 | JK340591 | 425 | putative beta-galactosidase [Oryza sativa Japonica Group] | BAD87855.1 | 8.00E-57 |
| CdL61 | JK340592 | 206 | gibberellin 20 oxidase 2 [Zea mays] | NP_001151167.1 | 7.00E-33 |
| CdL65 | JK340593 | 185 | diacylglycerol O-acyltransferase 1 [Zea mays] | ACG40288.1 | 1.00E-35 |
| CdL75 | JK340594 | 145 | 5,10-methylene-tetrahydrofolate reductase [Triticum monococcum] | ABJ15730.1 | 2.00E-16 |
| CdL85 | JK340595 | 280 | putative pyruvate dehydrogenase E1 alpha subunit [Oryza sativa Japonica Group] | BAD45661.1 | 1.00E-49 |
| CdL88 | JK340596 | 238 | purple acid phosphatase precursor [Zea mays] | ACG42188.1 | 5.00E-47 |
| CdL93 | JK340597 | 204 | ribonuclease 3 precursor [Oryza sativa (indica cultivar-group)] | ABR26024.1 | 0.004 |
| CdL96 | JK340598 | 196 | CER1 [Zea mays] | ACG47659.1 | 4.00E-12 |
| CdL69 | JK340599 | 362 | delta 1-pyrroline-5-carboxylate synthetase 1 [Sorghum bicolor] | ACU65226.1 | 5.00E-21 |
| CdL98 | JK340600 | 244 | delta 1-pyrroline-5-carboxylate synthase [Aeluropus lagopoides] | ABW05100.1 | 1.00E-44 |
| CdL105 | JK340601 | 225 | short-chain dehydrogenase, putative [Ricinus communis] | XP_002527853.1 | 7.00E-15 |
| CdL106 | JK340602 | 297 | serine esterase family protein [Zea mays] | ACG35252.1 | 3.00E-13 |
| CdL109 | JK340603 | 337 | putative acetyl-coenzyme A carboxylase ACC1A, [Oryza sativa (japonica cultivar-group)] | AAP53321.2 | 9.00E-59 |
| CdL112 | JK340604 | 154 | s-adenosylmethionine synthetase 1 mRNA[Oryza sativa (indica cultivar-group)] | EF576534.1 | 4.00E-60 |
|  |  |  |  |  |  |
|  |  |  | **osmoregulation** |  |  |
| CdL29 | JK340605 | 258 | putative UOS1 Predicted nucleoside-diphosphate-sugar epimerases [Oryza sativa Japonica Group] | BAD53796.1 | 1.00E-50 |
| CdL31 | JK340606 | 119 | galactinol synthase 3 [Zea mays] | ACG39512.1 | 3.00E-20 |
| CdL67 | JK340607 | 92 | galactinol synthase 1 [Zea mays] | AAQ07248 | 4.00E-14 |
| CdL68 | JK340608 | 290 | stachyose synthase [Zea mays] | NM_001154078.1 | 2.00E-11 |
|  |  |  |  |  |  |
|  |  |  | **membrane system** |  |  |
| CdL34 | JK340609 | 228 | leucine-rich repeat transmembrane protein kinase 1 [Zea mays] | AAC27894.1 | 1.00E-31 |
| CdL80 | JK340610 | 386 | Remorin, putative [Ricinus communis] | XP_002510796.1 | 5.00E-17 |
| CdL92 | JK340611 | 119 | membrane protein, putative, expressed [Oryza sativa (japonica cultivar-group)] | ABF93652.1 | 2.00E-06 |
| CdL94 | JK340612 | 184 | putative sodium-dicarboxylate cotransporter [Oryza sativa Japonica Group] | BAD09278.1 | 9.00E-09 |
|  |  |  |  |  |  |
|  |  |  | **signal and regulation** |  |  |
| CdL32 | JK340613 | 297 | putative CCAAT-binding transcription factor [Oryza sativa (japonicacultivar-group)] | ABF98349.1 | 0.016 |
| CdL33 | JK340614 | 165 | chitin-inducible gibberellin-responsive protein GRAS family transcription factor [Ricinus communis] | EEF39512.1 | 5.00E-06 |
| CdL39 | JK340588 | 239 | putative zinc finger protein [Oryza sativa Japonica Group] | AAS87371.1 | 3.00E-46 |
| CdL42 | JK340615 | 113 | EREBP-4 like protein [Zea mays] | EU954718.1 | 3.00E-11 |
| CdL64 | JK340616 | 111 | adenosine kinase 2 [Oryza sativa (indica cultivar-group)] | AAO72629.1 | 3.00E-16 |
| CdL73 | JK340617 | 781 | putative diacylglycerol kinase (with alternative splicing) [Oryza sativa (japonica cultivar-group)] | AAS07206.1 | 5.00E-137 |
| CdL79 | JK340618 | 104 | TPA_inf: WRKY transcription factor 50 [Oryza sativa (indica cultivar-group)] | DAA05116.1 | 9.00E-13 |
| CdL86 | JK340619 | 166 | serine kinase [Oryza sativa (indica cultivar-group)] | ABD72268.1 | 8.00E-27 |
| CdL100 | JK340620 | 101 | putative zinc finger and C2 domain protein [Oryza sativa Japonica Group] | BAD35474 | 1.00E-04 |
| CdL102 | JK340621 | 162 | SNF4 [Zea mays] | NP_001150464.1 | 1.00E-14 |
| CdL104 | JK340622 | 308 | serine/threonine-protein kinase SAPK8 [Zea mays] | NP_001149657.1 | 4.00E-58 |
|  |  |  |  |  |  |
|  |  |  | **structure protein** |  |  |
| CdL30 | JK340623 | 280 | putative nuclear matrix constituent protein 1 [Oryza sativa JaponicaGroup] | ABR25819.1 | 1.00E-21 |
| CdL72 | JK340624 | 106 | alpha tubulin-2A mRNA [Triticum aestivum] | DQ435667.1 | 2.00E-35 |
| CdL74 | JK340625 | 121 | actin depolymerization factor-like protein [Hordeum vulgare subsp.vulgare] | ACH70382.1 | 1.00E-14 |
|  |  |  |  |  |  |
|  |  |  | **protein synthesis and degradation** |  |  |
| CdL35 | JK340626 | 206 | ATP-dependent Clp protease ATP-binding subunit precursor [Oryza sativa (indica cultivar-group)] | AAN78327.1 | 6.00E-09 |
| CdL48 | JK340627 | 131 | ATP-dependent Clp protease proteolytic subunit 1 mRNA,[ Zea mays] | EU961934.1 | 1.00E-20 |
| CdL52 | JK340628 | 291 | protein phosphatase 2C [Triticum aestivum] | ABS11092.1 | 5.00E-38 |
| CdL57 | JK340629 | 206 | cysteine proteinase [Zea mays] | NP_001105479.1 | 6.00E-32 |
| CdL59 | JK340630 | 273 | eukaryotic peptide chain release factor subunit 1-1 [Zea mays] | NP_001147586.1 | 1.00E-07 |
| CdL63 | JK340630 | 255 | putative eukaryotic translation initiation factor 4G [Oryza sativa Japonica Group] | BAD30897.1 | 2.00E-15 |
| CdL76 | JK340631 | 128 | protein transport protein sec23, putative [Ricinus communis] | XP_002516399.1 | 7.00E-06 |
| CdL77 | JK340632 | 288 | Putative SKP1-like protein [Oryza sativa (japonica cultivar-group)] | AAN60492.1 | 4.00E-10 |
| CdL89 | JK340633 | 83 | proteasome subunit alpha type, putative [Ricinus communis] | XP_002513374.1 | 3.00E-09 |
| CdL91 | JK340634 | 125 | proteasome subunit beta type 7-A precursor [Zea mays] | ACG36096.1 | 2.00E-16 |
| CdL97 | JK340635 | 222 | putative 60S ribosomal protein L36 [Oryza sativa Japonica Group] | XP_002513689.1 | 1.00E-09 |
| CdL101 | JK340636 | 182 | ribosomal protein-like [Oryza sativa Japonica Group] | BAD36080.1 | 0.016 |
|  |  |  |  |  |  |
|  |  |  | **Energy** |  |  |
| CdL41 | JK340637 | 271 | thioredoxin H-type 5 [Zea mays] | NP_001158966.1 | 1.00E-05 |
| CdL55 | JK340638 | 76 | clone 245927 thioredoxin-like 1 mRNA [Zea mays] | EU962839.1 | 8.00E-07 |
| CdL113 | JK340639 | 261 | CTP synthase (LOC100281709), mRNA [Zea mays] | NM_001154629.1 | 5.00E-43 |
| CdL78 | JK340640 | 60 | soluble inorganic pyrophosphatase [Zea mays] | NP_001148294.1 | 0.002 |
|  |  |  |  |  |  |
|  |  |  | **other** |  |  |
| CdL47 | JK340641 | 163 | ATP-binding family protein [Arabidopsis thaliana] | NP_567393.1 | 7.00E-04 |
| CdL49 | JK340642 | 374 | LEC14B protein [Zea mays] | ACG43112.1 | 1.00E-69 |
| CdL50 | JK340643 | 140 | unknown [Zea mays] | ACR37600 | 9.00E-06 |
| CdL114 | JK340644 | 393 | Full=DEAD-box ATP-dependent RNA helicase 17 | RH17_ORYSJ | 3.00E-12 |
| CdL58 | JK340645 | 280 | hypothetical protein [Oryza sativa Japonica Group] | EAZ12481 | 8.00E-06 |
| CdL70 | JK340646 | 83 | hypothetical protein OsJ_18256 [Oryza sativa Japonica Group] | EEE63443.1 | 0.058 |
| CdL71 | JK340647 | 215 | nucleic acid binding protein [Zea mays] | ACG35732.1 | 9.00E-10 |
| CdL84 | JK340648 | 111 | pentatricopeptide, putative [Oryza sativa (japonica cultivar-group)] | AAP54520.1 | 4.00E-14 |
| CdL87 | JK340649 | 107 | hypothetical protein [Sorghum bicolor] | XP_002452785.1 | 3.00E-10 |
| CdL95 | JK340650 | 195 | RNA-binding protein 25 [Zea mays] | NP_001148482.1 | 7.00E-13 |
| CdL107 | JK340651 | 438 | RNA-binding protein of the Puf family,translational repressor [Translation, ribosomal structure and biogenesis] | XP_002516980.1 | 3.00E-17 |
| CdL108 | JK340652 | 217 | hypothetical protein, mRNA [Sorghum bicolor] | XM_002449071.1 | 6.00E-09 |

Table S4 BlastN (with the EST others database) analysis of ‘Tifway’ drought 5 days SSH library clones which had no match with reported genes or proteins

| Name | Accession  number | Length  (bp) | Gene identification | Similar gene accession  number | E-value |
| --- | --- | --- | --- | --- | --- |
| Tifway-5-42 | JK340653 | 42 | no significant homology |  |  |
| Tifway-5-43 | JK340654 | 48 | Bermudagrass Normalized cDNA [Cynodon dactylon ] | ES307217.1 | 3.00E-15 |
| Tifway-5-45 | JK340655 | 57 | no significant homology |  |  |
| Tifway-5-46 | JK340656 | 59 | no significant homology |  |  |
| Tifway-5-47 | JK340657 | 75 | no significant homology |  |  |
| Tifway-5-49 | JK340658 | 70 | FP834798 mixed stages (KT0AAA) Oikopleura dioica cDNA | FP834798.1 | 6.00E-13 |
| Tifway-5-50 | JK340659 | 75 | Bermudagrass cold acclimated cDNA[Cynodon dactylon ] | DN985680.1 | 2.00E-26 |
| Tifway-5-51 | JK340660 | 85 | Bermudagrass Normalized cDNA[Cynodon dactylon ] | ES299810.1 | 3.00E-31 |
| Tifway-5-52 | JK340661 | 78 | no significant homology |  |  |
| Tifway-5-53 | JK340662 | 85 | no significant homology |  |  |
| Tifway-5-54 | JK340663 | 84 | no significant homology |  |  |
| Tifway-5-56 | JK340664 | 95 | no significant homology |  |  |
| Tifway-5-57 | JK340665 | 80 | Potato tuber and Phytophthora infestans Phytophthora infestans cDNA | ES460190.1 | 5.00E-18 |
| Tifway-5-58 | JK340666 | 95 | JSYL507root-driving leaf SSH in rice [Oryza sativa Indica Group ] | CA752937.1 | 4.00E-24 |
| Tifway-5-60 | JK340667 | 105 | Bermudagrass Normalized cDNA[Cynodon dactylon ] | ES295032.1 | 9.00E-46 |
| Tifway-5-61 | JK340668 | 107 | Bermudagrass Normalized cDNA[Cynodon dactylon ] | ES303884.1 | 8.00E-47 |
| Tifway-5-63 | JK340669 | 116 | Bermudagrass Normalized cDNA[Cynodon dactylon ] | ES293251.1 | 5.00E-50 |
| Tifway-5-64 | JK340670 | 87 | no significant homology |  |  |
| Tifway-5-66 | JK340671 | 123 | no significant homology |  |  |
| Tifway-5-69 | JK340672 | 130 | no significant homology |  |  |
| Tifway-5-71 | JK340673 | 130 | Bermudagrass Normalized cDNA[Cynodon dactylon ] | ES296414.1 | 4.00E-14 |
| Tifway-5-72 | JK340674 | 137 | no significant homology |  |  |
| Tifway-5-73 | JK340675 | 132 | no significant homology |  |  |
| Tifway-5-74 | JK340676 | 134 | no significant homology |  |  |
| Tifway-5-75 | JK340677 | 143 | no significant homology |  |  |
| Tifway-5-76 | JK340678 | 147 | Festuca pratensis cDNA | GO894202.1 | 2.00E-32 |
| Tifway-5-77 | JK340679 | 158 | Bermudagrass Normalized cDNA[Cynodon dactylon ] | ES306020.1 | 4.00E-66 |
| Tifway-5-78 | JK340680 | 161 | Bermudagrass Normalized cDNA[Cynodon dactylon ] | ES305088.1 | 6.00E-76 |
| Tifway-5-79 | JK340681 | 168 | no significant homology |  |  |
| Tifway-5-80 | JK340682 | 166 | Bermudagrass Normalized cDNA[Cynodon dactylon | ES306021.1 | 2.00E-31 |
| Tifway-5-81 | JK340683 | 173 | Bermudagrass Normalized cDNA[Cynodon dactylon | ES297839.1 | 7.00E-63 |
| Tifway-5-82 | JK340684 | 178 | root cDNA of seedling 2ppm NAA(naphthalene acetic acid) [Oryza sativa Japonica Group] | CI560992.1 | 2.00E-12 |
| Tifway-5-83 | JK340685 | 180 | Maize Endosperm cDNA [Zea mays ] | CO469007.1 | 7.00E-70 |
| Tifway-5-84 | JK340686 | 179 | Bermudagrass Normalized cDNA[Cynodon dactylon ] | ES299486.1 | 1.00E-72 |
| Tifway-5-85 | JK340687 | 180 | Zea mays cDNA clone 997565 5', mRNA sequence | FL094996.1 | 9.00E-62 |
| Tifway-5-86 | JK340688 | 182 | Saccharum officinarum cDNA | CA263776.1 | 5.00E-21 |
| Tifway-5-88 | JK340689 | 191 | Bermudagrass Normalized cDNA[Cynodon dactylon ] | ES305201.1 | 2.00E-90 |
| Tifway-5-89 | JK340690 | 189 | no significant homology |  |  |
| Tifway-5-91 | JK340691 | 191 | Bermudagrass Normalized cDNA[Cynodon dactylon ] | ES306729.1 | 3.00E-31 |
| Tifway-5-92 | JK340692 | 196 | no significant homology |  |  |
| Tifway-5-93 | JK340693 | 199 | no significant homology |  |  |
| Tifway-5-94 | JK340694 | 205 | root salinity induced expressed sequence tag (EST) [Spartina alterniflora] | EH277904.1 | 2.00E-20 |
| Tifway-5-95 | JK340695 | 208 | no significant homology |  |  |
| Tifway-5-97 | JK340696 | 228 | OK-YZ-B139 Bermudagrass/SDS SSH Library Cynodon dactylon cDNA | BG322335.1 | 1.00E-105 |
| Tifway-5-98 | JK340697 | 233 | EC01 Eragrostis curvula cDNA | EH184804.1 | 6.00E-66 |
| Tifway-5-100 | JK340698 | 235 | Bermudagrass Normalized cDNA[Cynodon dactylon ] | DN985433.1 | 7.00E-97 |
| Tifway-5-101 | JK340699 | 238 | no significant homology |  |  |
| Tifway-5-102 | JK340700 | 245 | no significant homology |  |  |
| Tifway-5-103 | JK340701 | 255 | Bermudagrass cold acclimated cDNA [Cynodon dactylon ] | DN985546.1 | 1.00E-80 |
| Tifway-5-104 | JK340702 | 264 | CSCL5_C3_v5 Cleistogenes songorica drought stress induced library Cleistogenes songorica cDNA | JG394090.1 | 3.00E -89 |
| Tifway-5-106 | JK340703 | 271 | no significant homology |  |  |
| Tifway-5-108 | JK340704 | 310 | Bermudagrass Normalized cDNA[Cynodon dactylon ] | ES298097.1 | 2.00E-92 |
| Tifway-5-109 | JK340705 | 327 | no significant homology |  |  |
| Tifway-5-110 | JK340706 | 437 | Bermudagrass Normalized cDNA[Cynodon dactylon ] | ES300250.1 | 2.00E-36 |
| Tifway-5-111 | JK340707 | 445 | no significant homology |  |  |
|  |  |  |  |  |  |

| Name  Table S5 BlastX and BLastN analysis of ‘C299’ drought 10 days SSH library clones | Accession  number | Length  (bp) | Gene identification | Similar gene accession  number | E-value |
| --- | --- | --- | --- | --- | --- |
|  |  |  | **stress 、 defense and aging** |  |  |
| CdL10-18 | JK340519 | 208 | Zea mays clone 232597 heat shock 22 kDa protein mRNA, complete | EU961071.1 | 5.00E-10 |
| CdL10-19 | JK340520 | 110 | Zea mays clone 239755 peroxisomal carrier protein mRNA, complete cds | EU962036.1 | 9.00E-09 |
|  |  |  |  |  |  |
|  |  |  | **metabolism** |  |  |
| CdL10-4 | JK340521 | 199 | putative glutaryl-CoA dehydrogenase [Oryza sativa Japonica Group] | AAT07660.1 | 2.00E-36 |
| CdL10-11 | JK340522 | 324 | thioredoxin-like 1 [Zea mays] | ACG44900.1 | 2.00E-22 |
| CdL10-22 | JK340523 | 275 | isovaleryl-CoA dehydrogenase [Zea mays] | ACG40924.1 | 9.00E-13 |
| CdL10-26 | JK340524 | 274 | Sorghum bicolor aldehyde dehydrogenase (Dha1) mRNA, partial cds | U87982.1 | 3.00E-08 |
| CdL10-27 | JK340525 | 284 | plastid starch synthase I precursor [Zea mays] | NP_001104892.1 | 1.00E-36 |
| CdL10-30 | JK340526 | 270 | phosphosulfolactate synthase-related protein [Zea mays] | NP_001149731.1 | 1.00E-32 |
| CdL10-31 | JK340527 | 164 | MIOX2 (MYO-INOSITOL OXYGENASE 2); inositol oxygenase [Arabidopsisthaliana] | NP_565459.1 | 3.00E-26 |
| CdL10-36 | JK340528 | 209 | Dictyostelium discoideum AX4 cytochrome b5 reductase (DDB_G0278567)mRNA, complete cds | XM_637359.1 | 0.002 |
| CdL10-39 | JK340529 | 233 | Oryza sativa Indica Group cultivar Zhenshan 97B starch branching enzyme 4 (SBE4) gene, complete cds | GQ150940.1 | 2.00E-09 |
|  |  |  |  |  |  |
|  |  |  | **osmoregulation** |  |  |
| CdL10-20 | JK340530 | 147 | Zea mays clone 242635 galactinol synthase 3 mRNA, complete cds | EU962434.1 | 0.16 |
| CdL10-24 | JK340531 | 198 | Saccharum hybrid cultivar ROC22 sucrose transporter (SUT4) mRNA,complete cds | GQ485583.1 | 3.00E-06 |
|  |  |  |  |  |  |
|  |  |  | **membrane system** |  |  |
|  |  |  |  |  |  |
|  |  |  | **proteins synthetise and degradation** |  |  |
| CdL10-28 | JK340532 | 227 | eukaryotic peptide chain release factor subunit 1-1 [Zea mays] | NP_001147586.1 | 4.00E-37 |
| CdL10-41 | JK340533 | 219 | Arabidopsis thaliana aminopeptidase (APM1) mRNA | NM_119463.4 | 0.006 |
|  |  |  |  |  |  |
|  |  |  | **signals and regulation** |  |  |
| CdL10-8 | JK340534 | 216 | protein usf [Oryza sativa Indica Group] | ABR25516.1 | 2.00E-29 |
| CdL10-10 | JK340535 | 155 | choline/ethanolamine kinase [Zea mays] | ACG32241.1 | 2.00E-05 |
| CdL10-12 | JK340536 | 148 | CBL-interacting protein kinase 30 [Sorghum bicolor] | ACQ83502.1 | 8.00E-19 |
| CdL10-23 | JK340537 | 226 | Zea mays clone 330462 ATPase inhibitor mRNA, complete cds | EU969453.1 | 3.00E-13 |
| CdL10-29 | JK340538 | 263 | ethylene-responsive protein [Zea mays] | NP_001148498.1 | 9.00E-39 |
| CdL10-38 | JK340539 | 266 | RecName: Full=Homeobox protein knotted-1-like 2; AltName: Full=Homeobox protein HOS58 | Q0E3C3.2|KNOS2_ORYSJ | 9.00E-44 |
| CdL10-7 | JK340540 | 302 | guanylyl cyclase [Triticum aestivum] | ABD18449.1 | 3.00E-46 |
|  |  |  |  |  |  |
|  |  |  | **energy** |  |  |
| CdL10-2 | JK340541 | 183 | putative ATPase [Oryza sativa Japonica Group] | BAD52846.1 | 6.00E-08 |
|  |  |  |  |  |  |
|  |  |  | **others** |  |  |
| CdL10-15 | JK340542 | 206 | Triticum aestivum Ran-binding protein mRNA, complete cds | DQ109671.1 | 8.00E-39 |
| CdL10-25 | JK340543 | 221 | Plasmodium yoelii rhoptry protein gene, complete cds | U36927.3 | 4.00E-05 |
| CdL10-37 | JK340544 | 284 | RecName: Full=Two-component response regulator-like PRR73; AltName: Full=Pseudo-response regulator 73; Short=OsPRR73 | A2XFB7.2 | 3.00E-08 |
| CdL10-21 | JK340545 | 246 | Zea mays clone 291710 ML domain protein mRNA, complete cds | EU966097.1 | 1.00E-18 |
| CdL10-5 | JK340546 | 198 | expressed protein [Oryza sativa (japonica cultivar-group)] | ABA98709.1 | 7.00E-14 |
| CdL10-16 | JK340547 | 224 | Chaperonin-like RbcX protein [Arabidopsis thaliana] | NP_568382.1 | 1.00E-26 |
| CdL10-17 | JK340548 | 338 | hypothetical protein OsI_25487 [Oryza sativa Indica Group] | EEC81785.1 | 5.00E-20 |
| CdL10-32 | JK340549 | 130 | Sorghum bicolor hypothetical protein, mRNA | XM_002438260.1 | 1.00E-14 |
| CdL10-33 | JK340550 | 262 | Oryza sativa Japonica Group cDNA clone:J033145L05, full insert sequence [Oryza sativa Japonica Group] | AK103790.1 | 7.00E-16 |
| CdL10-34 | JK340551 | 169 | Sorghum bicolor hypothetical protein, mRNA [Sorghum bicolor] | XM_002458915.1 | 0.47 |
| CdL10-40 | JK340552 | 266 | Zea mays hypothetical protein LOC100193803 (LOC100193803), mRNA | NM_001138887.1 | 0.092 |

| Name  Table S6 BlastN (with the EST others database) analysis of ‘C299’ drought 10 days SSH library clones which had no match with reported genes or proteins | Accession  number | Length  (bp) | Gene identification | Similar gene accession  number | E-value |
| --- | --- | --- | --- | --- | --- |
| C299-10-1 | JK340553 | 207 | EDR-151 Eleusine coracana water deficit stress induced cDNA similar to late embryogenesis abundant protein | FD661937.1 | 3.00E-18 |
| C299-10-2 | JK340554 | 185 | Panicum virgatum late flowering buds + seed development (L) Panicum virgatum cDNA clone CCGH25609 5', mRNA sequence. | FL846929.1 | 6.00E-14 |
| C299-10-3 | JK340555 | 140 | wpi2s.pk006.m2 wpi2s Triticum aestivum cDNA clone wpi2s.pk006.m2 5' end | CA738225.1 | 7.00E-06 |
| C299-10-4 | JK340556 | 206 | root4_d14.y1.abd tef (Kaye Murri) root Eragrostis tef cDNA clone root4_d14.y1.abd | DN483160.1 | 6.00E-08 |
| C299-10-5 | JK340557 | 244 | MSU28_F_PLATE_3_C12 Bermudagrass cv. MSU subtracted cold acclimated cDNA library Cynodon dactylon cDNA clone | DN985598.1 | 7.00E-59 |
| C299-10-7 | JK340558 | 252 | MSU2_F_PLATE_2_C02 Bermudagrass cv. MSU subtracted cold acclimated cDNA library Cynodon dactylon cDNA clone | DN985663.1 | 4.00E-11 |
| C299-10-8 | JK340559 | 192 | MSU28_F_PLATE_2_C02 Bermudagrass cv. MSU subtracted cold acclimated cDNA library Cynodon dactylon cDNA | DN985762.1 | 1.00E-10 |
| C299-10-9 | JK340560 | 195 | MSU28_F_PLATE_4_H03 Bermudagrass cv. MSU subtracted cold acclimated cDNA library Cynodon dactylon cDNA clone | DN985763.1 | 4.00E-86 |
| C299-10-9(c) | JK340575 | 180 | MSU2_F_PLATE_3_A08 Bermudagrass cv. MSU subtracted cold acclimated cDNA library Cynodon dactylon cDNA clone MSU_2F_2-II_03_MSU_2F_2-II_03_A08.ab1, mRNA sequence. | DN986005.1 | 2.00E-83 |
| C299-10-12 | JK340561 | 215 | ZEBRA2_F_PLATE_5_C05 Bermudagrass line Zebra subtracted cold acclimated cDNA library Cynodon dactylon cDNA clone | DN987418.1 | 9.00E-57 |
| C299-10-13 | JK340562 | 162 | PsSamTSujD88 SSH-derived Papaver somniferum genotype Sampada-specific capsule wall cDNA library Papaver somniferum cDNA clone PsSamTSujD88, mRNA sequence. | GT571582.1 | 4.00E-34 |
| C299-10-13(b) | JK340576 | 180 | ZEBRA2_F_PLATE_2_F02 Bermudagrass line Zebra subtracted cold acclimated cDNA library Cynodon dactylon cDNA clone ZEBRA_2F_2-II_02_ZEBRA_2F_2-II_02_F02.ab1, mRNA sequence. | DN987485.1 | 9.00E-37 |
| C299-10-14 | JK340563 | 213 | Synthetic construct Saccharomyces cerevisiae clone FLH148239.01X BRO1 gene | DQ332224.1 | 4.00E-05 |
| C299-10-15 | JK340564 | 124 | LRAGE048411 Liver regeneration after partial hepatectomy Rattus norvegicus cDNA | DW335447.1 | 6.00E-06 |
| C299-10-17 | JK340565 | 156 | Bermudagrass Normalized cDNA Library Cynodon dactylon cDNA 5', | ES293182.1 | 1.00E-15 |
| C299-10-19 | JK340566 | 210 | Bermudagrass Normalized cDNA Library Cynodon dactylon M65cDNA 5' | gb|ES304340.1| | 2.00E-84 |
| C299-10-20 | JK340567 | 243 | Bermudagrass Normalized cDNA Library Cynodon dactylon cDNA 5' mRNA sequence | ES306690.1 | 3.00E-69 |
| C299-10-28 | JK340568 | 302 | no significant homology |  |  |
| C299-10-29 | JK340569 | 146 | no significant homology |  |  |
| C299-10-31 | JK340570 | 282 | no significant homology |  |  |
| C299-10-32 | JK340571 | 256 | no significant homology |  |  |
| C299-10-33 | JK340572 | 301 | no significant homology |  |  |
| C299-10-35 | JK340573 | 192 | no significant homology |  |  |
| C299-10-37 | JK340574 | 115 | no significant homology |  |  |
|  |  |  |  |  |  |

| Name  Table S7 BlastX and BLastN analysis of ‘Tifway’ drought 10 days SSH library clones | Accession  number | Length  (bp) | Gene identification | Similar gene  accession  number | E-value |
| --- | --- | --- | --- | --- | --- |
|  |  |  | **stress, defense and aging** |  |  |
| CdL10-52 | JK340708 | 371 | putative superoxide dismutase [Cu-Zn], chloroplast precursor [Oryza sativa Japonica Group] | BAD09607.1 | 2.00E-60 |
| CdL10-53 | JK340709 | 221 | putative stress related-like protein interactor [Oryza sativa Japonica Group] | BAD09693.1 | 5.00E-28 |
| CdL10-57 | JK340710 | 515 | putative disease resistance protein I2 [Oryza sativa Japonica Group] | BAD22413.1 | 5.00E-22 |
| CdL10-84 | JK340711 | 470 | putative peroxisome assembly protein 2 [Oryza sativa Japonica Group] | AAT85132.1 | 9.00E-93 |
| CdL10-96 | JK340712 | 227 | bifunctional nuclease in basal defense response [Oryza minuta] | ABI79452.1 | 1.00E-28 |
| CdL10-98 | JK340713 | 289 | putative senescence-associated protein [Lilium longiflorum] | ABO20851.1 | 3.00E-58 |
| CdL10-99 | JK340714 | 185 | 2-cys peroxiredoxin bas1 [Oryza sativa Indica Group] | ABR25513.1 | 1.00E-33 |
| CdL10-100 | JK340715 | 321 | disease resistance protein rpm1 [Oryza sativa Indica Group] | ABR25813.1 | 7.00E-27 |
| CdL10-114 | JK340716 | 378 | glutathione peroxidase 4 [Zea mays] | ACG32415.1 | 7.00E-08 |
| CdL10-128 | JK340717 | 190 | putative heat shock factor binding protein [Oryza officinalis] | ADD60691.1 | 7.00E-13 |
| CdL10-131 | JK340718 | 314 | Cynodon dactylon metallothionein-like protein mRNA, complete | AY574281.1 | 3.00E-66 |
| CdL10-149 | JK340719 | 417 | dnaJ (DnaJ/Hsp40 (heat shock protein 40) proteins)domain containing protein [Zea mays] | NP_001149610.1 | 8.00E-33 |
|  |  |  |  |  |  |
|  |  |  | **metabolism** |  |  |
| CdL10-47 | JK340720 | 413 | putative aldehyde oxidase [Oryza sativa Japonica Group] | BAC84232.1 | 9.00E-46 |
| CdL10-50 | JK340721 | 461 | putative glycoprotein 3-alpha-L-fucosyltransferase [Oryza sativa Japonica Group] | BAD09365.1 | 4.00E-74 |
| CdL10-59 | JK340722 | 217 | putative acetyl-CoA synthetase [Oryza sativa Japonica Group] | BAD25398.1 | 3.00E-36 |
| CdL10-60 | JK340723 | 396 | putative ribulokinase [Oryza sativa Japonica Group] | BAD27990.1 | 2.00E-68 |
| CdL10-61 | JK340724 | 238 | putative serine decarboxylase [Oryza sativa Japonica Group] | BAD28070.1 | 1.00E-43 |
| CdL10-64 | JK340725 | 516 | putative tDET1 protein [Oryza sativa Japonica Group] | BAD44839.1 | 1.00E-104 |
| CdL10-67 | JK340726 | 173 | ferredoxin-dependent glutamate synthase precursor [Oryza sativa] | BAF46922.1 | 6.00E-17 |
| CdL10-69 | JK340727 | 59 | photosystem q(b) protein [Arthrospira platensis str. Paraca] | ZP_06385206.1 | 2.00E-05 |
| CdL10-79 | JK340728 | 443 | glycine dehydrogenase P protein [Oryza sativa Japonica Group] | AAQ24377.1 | 4.00E-82 |
| CdL10-82 | JK340729 | 319 | putative malate dehydrogenase [Oryza sativa Japonica Group] | AAT69584.1 | 3.00E-05 |
| CdL10-83 | JK340730 | 221 | cell-wall invertase 4 [Oryza sativa Japonica Group] | AAT84404.1 | 1.00E-35 |
| CdL10-85 | JK340731 | 164 | hydrolase, alpha/beta fold family protein, expressed [Oryza sativa (japonica cultivar-group)] | ABA91184.1 | 5.00E-21 |
| CdL10-88 | JK340732 | 280 | Nodulin-like family protein, expressed [Oryza sativa (japonica cultivar-group)] | ABA95619.1 | 4.00E-24 |
| CdL10-89 | JK340733 | 269 | SET domain containing protein (Rubisco LSMT substrate-binding), expressed [Oryza sativa (japonica cultivar-group)] | ABA96840.1 | 2.00E-38 |
| CdL10-92 | JK340734 | 365 | Alpha-L-arabinofuranosidase C-terminus family protein, expressed [Oryza sativa (japonica cultivar-group)] | ABF95656.1 | 8.00E-62 |
| CdL10-93 | JK340735 | 243 | Hydroxyacylglutathione hydrolase, putative, expressed [Oryza sativa (japonica cultivar-group)] | ABF95772.1 | 7.00E-49 |
| CdL10-95 | JK340736 | 347 | acyl-CoA binding family protein, putative, expressed [Oryza sativa (japonica cultivar-group)] | ABF99749.1 | 2.00E-36 |
| CdL10-97 | JK340737 | 134 | glyceraldehyde-3-phosphate dehydrogenase [Urochloa decumbens] | ABK96988.1 | 5.00E-09 |
| CdL10-103 | JK340738 | 208 | delta-1-pyrroline-5-carboxylate synthetase [Saccharum officinarum] | ABS32296.2 | 1.00E-16 |
| CdL10-106 | JK340739 | 364 | aspartate aminotransferase [Oryza granulata] | ABY68128.1 | 6.00E-26 |
| CdL10-109 | JK340740 | 166 | glutamine amidotransferase subunit pdxT [Zea mays] | ACG26547.1 | 7.00E-20 |
| CdL10-117 | JK340741 | 661 | UDP-glucose 4-epimerase GEPI48 [Zea mays] | ACG36581.1 | 2.00E-47 |
| CdL10-118 | JK340742 | 205 | isovaleryl-CoA dehydrogenase [Zea mays] | ACG40924.1 | 2.00E-34 |
| CdL10-120 | JK340743 | 340 | nudix hydrolase 2 [Zea mays] | ACG43116.1 | 2.00E-15 |
| CdL10-121 | JK340744 | 231 | nuclease | ACG43533.1 | 5.00E-37 |
| CdL10-123 | JK340745 | 475 | 9,10-9,10 carotenoid cleavage dioxygenase 1 [Zea mays] | ACG46084.1 | 3.00E-66 |
| CdL10-125 | JK340746 | 137 | formamidase [Lupinus albus] | ACM68705.1 | 2.00E-21 |
| CdL10-127 | JK340747 | 364 | dehydroascorbate reductase [Triticum aestivum] | ACV89491.1 | 9.00E-47 |
| CdL10-135 | JK340748 | 258 | Chain A, Ketol-Acid Reductoisomerase (Kari) In Complex With Mg2+ | 3FR7|A | 3.00E-27 |
| CdL10-143 | JK340749 | 403 | core alpha 1,3-fucosyltransferase [Zea mays] | NP_001105927.1 | 5.00E-58 |
| CdL10-145 | JK340750 | 294 | purple acid phosphatase [Zea mays] | NP_001147979.1 | 2.00E-60 |
| CdL10-146 | JK340751 | 214 | aminoacylase-1 [Zea mays] | NP_001148589.1 | 6.00E-08 |
| CdL10-147 | JK340752 | 443 | aldehyde dehydrogenase family 7 member A1 [Zea mays] | NP_001149126.1 | 5.00E-75 |
| CdL10-152 | JK340753 | 174 | succinyl-CoA ligase beta-chain [Zea mays] | NP_001150653.1 | 4.00E-27 |
| CdL10-154 | JK340754 | 156 | angustifolia (Rossmann-fold NAD(P)(+)-binding proteins) [Zea mays] | NP_001151564.1 | 3.00E-06 |
| CdL10-155 | JK340755 | 308 | rieske domain containing protein [Zea mays] | NP_001151666.1 | 4.00E-07 |
|  |  |  |  |  |  |
|  |  |  | **membrane system** |  |  |
| CdL10-42 | JK340756 | 132 | putative triose phosphate/phosphate translocator [Oryza sativa Japonica Group] | BAB17213.1 | 1.00E-18 |
| CdL10-48 | JK340757 | 452 | putative membrane related protein CP5 [Oryza sativa Japonica Group] | BAD07966.1 | 3.00E-89 |
| CdL10-90 | JK340758 | 509 | Phospholipid-transporting ATPase 3, putative, expressed [Oryza sativa (japonica cultivar-group)] | ABB47597.2 | 4.00E-108 |
| CdL10-112 | JK340759 | 339 | protein translocase/ protein transporter [Zea mays] | ACG31334.1 | 3.00E-22 |
| CdL10-119 | JK340760 | 168 | vacuolar sorting receptor 1 precursor [Zea mays] | ACG42879.1 | 6.00E-27 |
| CdL10-124 | JK340761 | 417 | SNARE domain containing protein [Zea mays] | ACG48507.1 | 8.00E-81 |
| CdL10-133 | JK340762 | 108 | Oryza sativa (indica cultivar-group) cultivar FL478 putative vacuolar assembly protein VPS41 genomic sequence | EF589297.1 | 6.00E-11 |
| CdL10-139 | JK340763 | 320 | Zea mays vacuolar sorting receptor 7 (LOC100286125), mRNA | NM_001159013.1 | 9.00E-16 |
| CdL10-141 | JK340764 | 749 | RecName: Full=Probable metal-nicotianamine transporter YSL6; AltName: Full=Protein YELLOW STRIPE LIKE 6; Short=OsYSL6 | Q7XRV2.1 | 4.00E-160 |
| CdL10-153 | JK340765 | 279 | transmembrane 9 superfamily protein member 1 [Zea mays] | NP_001151315.1 | 2.00E-12 |
| CdL10-167 | JK340766 | 339 | oligopeptide transporter OPT family [Populus trichocarpa] | XP_002329927.1 | 1.00E-11 |
|  |  |  |  |  |  |
|  |  |  | **signal and regulation** |  |  |
| CdL10-43 | JK340767 | 485 | bZIP transcription factor [Oryza sativa] | BAB72062.1 | 4.00E-45 |
| CdL10-45 | JK340768 | 232 | casein kinase I-like [Oryza sativa Japonica Group] | BAB92346.1 | 6.00E-46 |
| CdL10-46 | JK340769 | 96 | putative calreticulin [Oryza sativa Japonica Group] | BAC06263.1 | 1.00E-13 |
| CdL10-54 | JK340770 | 420 | putative calmodulin-binding protein [Oryza sativa Japonica Group] | BAD16554.1 | 2.00E-59 |
| CdL10-55 | JK340771 | 576 | putative protein kinase Xa21, receptor type precursor [Oryza sativa Japonica Group] | BAD19467.1 | 7.00E-39 |
| CdL10-63 | JK340772 | 520 | PHD finger protein-like [Oryza sativa Japonica Group] | BAD35905.1 | 7.00E-11 |
| CdL10-65 | JK340773 | 191 | putative receptor protein kinase PERK1 [Oryza sativa Japonica Group] | BAD67605.1 | 1.00E-28 |
| CdL10-68 | JK340774 | 297 | casein kinase II alpha subunit [Oryza brachyantha] | BAG82869.1 | 6.00E-44 |
| CdL10-71 | JK340775 | 251 | putative phospholipase D [Hordeum vulgare subsp. vulgare] | CAD42652.1 | 7.00E-52 |
| CdL10-72 | JK340776 | 635 | MIKC-type MADS-box transcription factor WM22B [Triticum aestivum] | CAM59068.1 | 4.00E-78 |
| CdL10-86 | JK340777 | 270 | EF hand family protein, expressed [Oryza sativa (japonica cultivar-group)] | ABA91405.1 | 4.00E-19 |
| CdL10-87 | JK340778 | 294 | ETO1((ETHYLENE OVERPRODUCER 1)-like protein 1, putative, expressed [Oryza sativa (japonica cultivar-group)] | ABA94447.1 | 8.00E-34 |
| CdL10-104 | JK340779 | 199 | lammer-type protein kinase [Sorghum bicolor] | BU88852.1 | 1.00E-14 |
| CdL10-113 | JK340780 | 214 | protein usf [Zea mays](Dienelactone hydrolase family; pfam01738) | ACG32282.1 | 1.00E-42 |
| CdL10-105 | JK340781 | 399 | serine/threonine protein kinase [Dasypyrum villosum] | ABV81083.1 | 5.00E-22 |
| CdL10-107 | JK340782 | 293 | SKIP interacting protein 25 [Oryza sativa] | ACA64838.1 | 1.00E-13 |
| CdL10-111 | JK340783 | 391 | serine/threonine protein phosphatase 2A 55 kDa regulatory subunit Bbeta isoform [Zea mays] | ACG29079.1 | 2.00E-68 |
| CdL10-130 | JK340784 | 183 | Oryza sativa chitin-inducible gibberellin-responsive protein (CIGR1) mRNA, complete cds | AY062209.1 | 9.00E-19 |
| CdL10-159 | JK340785 | 310 | IRE1A; endoribonuclease/ kinase [Arabidopsis thaliana] | NP_565419.1 | 3.00E-18 |
| CdL10-177 | JK340786 | 191 | Gibberellin 20 oxidase, putative [Ricinus communis] | XP_002517541.1 | 4.00E-05 |
| CdL10-180 | JK340787 | 419 | RecName: Full=Zinc finger CCCH domain-containing protein 27; Short=OsC3H27 | Q0JDM0.2 | 4.00E-45 |
| CdL10-182 | JK340788 | 256 | RecName: Full=Cyclin-dependent kinase F-4; Short=CDKF;4; AltName: Full=Serine/threonine-protein kinase MHK-like protein 2 | Q6Z8C8.1 | 1.00E-28 |
| CdL10-183 | JK340789 | 479 | RecName: Full=Zinc finger CCCH domain-containing protein 50; Short=OsC3H50; AltName: Full=Protein ZF | Q84SL2.1 | 2.00E-29 |
|  |  |  |  |  |  |
|  |  |  | **structure protein** |  |  |
| CdL10-58 | JK340790 | 528 | putative Golgi-associated particle 102K chain [Oryza sativa Japonica Group] | BAD25013.1 | 4.00E-21 |
| CdL10-66 | JK340791 | 446 | putative MAR binding filament-like protein 1 [Oryza sativa Japonica Group] | BAD68082.1 | 7.00E-62 |
| CdL10-94 | JK340792 | 541 | unconventional myosin heavy chain, putative, expressed [Oryza sativa (japonica cultivar-group)] | ABF98259.1 | 2.00E-97 |
| CdL10-129 | JK340793 | 490 | Porteresia coarctata histone H3 mRNA, complete cds | AF109910.1 | 4.00E-105 |
| CdL10-142 | JK340794 | 222 | RecName: Full=Protein HIRA; AltName: Full=Histone regulator protein | NP_001063964.1 | 2.00E-42 |
|  |  |  |  |  |  |
|  |  |  | **protein synthesis and degradation** |  |  |
| CdL10-44 | JK340795 | 406 | F-box protein-like [Oryza sativa Japonica Group] | BAB90562.1 | 5.00E-59 |
| CdL10-51 | JK340796 | 546 | putative RNA recognition motif (RRM)-containing protein [Oryza sativa Japonica Group] | BAD09516.1 | 4.00E-81 |
| CdL10-56 | JK340797 | 210 | putative cysteine proteinase 1 precursor [Oryza sativa Japonica Group] | BAD19579.1 | 5.00E-39 |
| CdL10-70 | JK340798 | 455 | putative WD-repeat protein [Oryza sativa] | CAD29285.1 | 6.00E-24 |
| CdL10-73 | JK340799 | 453 | putative ribosomal protein S3 [Vigna unguiculata] | CAO02550.1 | 5.00E-27 |
| CdL10-75 | JK340800 | 469 | putative component of a tRNA splicing complex [Oryza sativa Japonica Group] | AAO23082.1 | 6.00E-95 |
| CdL10-76 | JK340801 | 226 | Putative Transcription initiation factor IIE, beta subunit [Oryza sativa Japonica Group] | AAP53614.1 | 8.00E-39 |
| CdL10-77 | JK340802 | 175 | transcriptional regulator, putative, expressed [Oryza sativa(japonica cultivar-group)] | AAP54975.2 | 8.00E-28 |
| CdL10-91 | JK340803 | 162 | kelch repeat-containing F-box family protein, putative, expressed [Oryza sativa (japonica cultivar-group)] | ABF94218.1 | 9.00E-26 |
| CdL10-101 | JK340804 | 174 | 60S ribosomal protein l22-2 [Oryza sativa Indica Group] | ABR25829.1 | 5.00E-20 |
| CdL10-108 | JK340805 | 316 | elongin C [Zea mays] | ACG26421.1 | 7.00E-17 |
| CdL10-110 | JK340806 | 516 | mediator of RNA polymerase II transcription subunit 18 [Zea mays] | ACG28268.1 | 2.00E-55 |
| CdL10-115 | JK340807 | 366 | thiol protease aleurain precursor [Zea mays] | ACG34091.1 | 8.00E-46 |
| CdL10-116 | JK340808 | 450 | seryl-tRNA synthetase [Zea mays] | ACG34450.1 | 1.00E-24 |
| CdL10-122 | JK340809 | 101 | 60S ribosomal protein L30 [Zea mays] | ACG46009.1 | 4.00E-15 |
| CdL10-126 | JK340810 | 202 | appr-1-p (ADP-ribose-1"-monophosphate) processing enzyme family protein [Sonneratia alba] | ACS68709.1 | 3.00E-14 |
| CdL10-132 | JK340811 | 220 | Oryza sativa (indica cultivar-group) clone V-H10 ubiquitin fusion protein mRNA, partial cds | EF576142.1 | 4.00E-18 |
| CdL10-134 | JK340812 | 346 | CTD-phosphatase-like protein [Zea mays] | NP_001149415.1 | 9.00E-38 |
| CdL10-138 | JK340813 | 337 | Zea mays dolichyl-diphosphooligosaccharide --protein glycosyltransferase 67 kDasubunit (LOC100282884), mRNA | NM_001155790.1 | 7.00E-49 |
| CdL10-140 | JK340814 | 327 | Zea mays USP(the ubiquitin-specific processing protease (USP/UBP) superfamily) family protein (LOC100304112), mRNA | NM_001165595.1 | 3.00E-16 |
| CdL10-148 | JK340815 | 254 | 60S ribosomal protein L10-3 [Zea mays] | NP_001149336.1 | 2.00E-11 |
| CdL10-156 | JK340816 | 386 | ubiquitin ligase protein COP1 [Zea mays] | NP_001152482.1 | 6.00E-73 |
| CdL10-157 | JK340817 | 199 | 112 kDa replicase protein [Panicum mosaic virus] | NP_068342.1 | 2.00E-23 |
| CdL10-158 | JK340818 | 328 | ATPREP1 (PRESEQUENCE PROTEASE 1); metalloendopeptidase [Arabidopsis thaliana] | NP_188548.2 | 2.00E-41 |
| CdL10-176 | JK340819 | 400 | eukaryotic translation elongation factor, putative [Ricinus communis] | XP_002513404.1 | 2.00E-40 |
| CdL10-178 | JK340820 | 280 | ubiquitin specific protease 39 and snrnp assembly factor, putative [Ricinus communis] | XP_002520562.1 | 7.00E-41 |
| CdL10-179 | JK340821 | 420 | RNA polymerase II ctd phosphatase, putative [Ricinus communis] | XP_002526210.1 | 9.00E-32 |
| CdL10-181 | JK340822 | 370 | RecName: Full=Splicing factor U2af (Splicing factor ) large subunit B; AltName: Full=U2 small nuclear ribonucleoprotein auxiliary factor large subunit B; Short=U2 snRNP auxiliary factor large subunit B; AltName: Full=U2 auxiliary factor 65 kDa subunit B | Q2QKB4.1 | 3.00E-30 |
|  |  |  |  |  |  |
|  |  |  | **Energy** |  |  |
| CdL10-41 (Hyb) | JK340916 | 279 | AAA-type ATPase-like protein [Oryza sativa Japonica Group] | BAC56025.1 | 2.00E-05 |
| CdL10-62 | JK340823 | 630 | putative mitochondrial energy transfer protein [Oryza sativa Japonica Group] | BAD35459.1 | 1.00E-122 |
| CdL10-81 | JK340824 | 503 | putative GTP-binding protein [Oryza sativa Japonica Group] | AAT39172.1 | 8.00E-83 |
| CdL10-151 | JK340825 | 565 | nucleolar GTP-binding protein 2 [Zea mays] | NP_001150224.1 | 2.00E-110 |
|  |  |  |  |  |  |
|  |  |  | **other** |  |  |
| CdL10-74 | JK340826 | 436 | putative ATP-dependent RNA helicase (5'-partial) [Oryza sativa Japonica Group] | AAG21915.1 | 2.00E-79 |
| CdL10-78 | JK340827 | 194 | multifunctional protein | AAQ13901.1 | 3.00E-25 |
| CdL10-80 | JK340828 | 559 | Mlo3 [Hordeum vulgare subsp. vulgare] | AAS93431.1 | 5.00E-87 |
| CdL10-102(a) | JK340829 | 436 | retrotransposon protein [Oryza sativa Indica Group] | ABR26094.1 | 3.00E-18 |
| CdL10-102(b) | JK340830 | 302 | retrotransposon protein [Oryza sativa Indica Group] | ABR26094.1 | 7.00E-32 |
| CdL10-137 | JK340831 | 389 | Zea mays protein BRE (invovle in DNA repair)(LOC100282640), mRNA | NM_001155548.1 | 2.00E-19 |
| CdL10-150 | JK340832 | 392 | seed maturation protein [Zea mays] | NP_001149653.1 | 9.00E-72 |
| CdL10-49 | JK340833 | 684 | unknown protein [Oryza sativa Japonica Group] | BAD09002.1 | 2.00E-25 |
| CdL10-144 | JK340834 | 168 | nifU-like N-terminal domain-containing protein [Zea mays] | NP_001146933.1 | 4.00E-16 |
| CdL10-160 | JK340835 | 141 | Sorghum bicolor hypothetical protein, mRNA | XM_002443118.1 | 2.00E-07 |
| CdL10-161 | JK340836 | 168 | Sorghum bicolor hypothetical protein, mRNA | XM_002452072.1 | 3.00E-17 |
| CdL10-162 | JK340837 | 450 | Sorghum bicolor hypothetical protein, mRNA | XM_002452962.1 | 1.00E-79 |
| CdL10-163 | JK340838 | 323 | Sorghum bicolor hypothetical protein, mRNA | XM_002455421.1 | 8.00E-23 |
| CdL10-164 | JK340839 | 175 | Sorghum bicolor hypothetical protein, mRNA | XM_002460071.1 | 8.00E-13 |
| CdL10-165 | JK340840 | 98 | Sorghum bicolor hypothetical protein, mRNA | XM_002464009.1 | 4.00E-06 |
| CdL10-166 | JK340841 | 246 | Sorghum bicolor hypothetical protein, mRNA | XM_002466964.1 | 3.00E-14 |
| CdL10-168 | JK340842 | 214 | hypothetical protein SORBIDRAFT_10g006740 [Sorghum bicolor] | XP_002436662.1 | 5.00E-20 |
| CdL10-169 | JK340843 | 358 | hypothetical protein SORBIDRAFT_10g002430 [Sorghum bicolor] | XP_002437780.1 | 4.00E-25 |
| CdL10-170 | JK340844 | 191 | hypothetical protein SORBIDRAFT_07g000660 [Sorghum bicolor] | XP_002443709.1 | 1.00E-22 |
| CdL10-171 | JK340845 | 139 | hypothetical protein SORBIDRAFT_04g022140 [Sorghum bicolor] | XP_002452231.1 | 1.00E-06 |
| CdL10-172 | JK340846 | 365 | hypothetical protein SORBIDRAFT_03g004445 [Sorghum bicolor] | XP_002455107.1 | 2.00E-66 |
| CdL10-173 | JK340847 | 335 | Rossmann-fold NAD(P)-binding domain-containing protein [Arabidopsis thaliana] | NP_181441.3 | 3.00E-49 |
| CdL10-174 | JK340848 | 152 | hypothetical protein SORBIDRAFT_02g005870 [Sorghum bicolor] | XP_002461646.1 | 1.00E-11 |
| CdL10-175 | JK340849 | 517 | hypothetical protein SORBIDRAFT_01g017460 [Sorghum bicolor] | XP_002466963.1 | 2.00E-62 |
| CdL10-184 | JK340850 | 128 | hypothetical protein OsJ_18363 [Oryza sativa Japonica Group] | EEE63547.1 | 4.00E-18 |
|  |  |  |  |  |  |

| Name  Table S8 BlastN (with the EST others database) analysis of ‘Tifway’ drought 10d SSH library clones which had no match with reported genes or proteins | Accession  number | Length  (bp) | Gene identification | Similar gene accession  number | E-value |
| --- | --- | --- | --- | --- | --- |
| Tifway-10-39 | JK340851 | 151 | Bermudagrass Normalized cDNA Library Cynodon dactylon cDNA 5' | ES298643.1 | 2.00E-70 |
| Tifway-10-40 | JK340852 | 237 | Bermudagrass Normalized cDNA Library Cynodon dactylon cDNA 5' | ES303344.1 | 7.00E-08 |
| Tifway-10-41 | JK340853 | 111 | CT846599 Oryza sativa (indica cultivar-group) cv. Guang Lu Ai 4 Oryza sativa Indica Group cDNA clone CONTIG5372, mRNA sequence. | CT846599.1 | 5.00E-18 |
| Tifway-10-43 | JK340854 | 331 | Bermudagrass Normalized cDNA Library Cynodon dactylon cDNA 5', | ES295356.1 | 1.00E-19 |
| Tifway-10-45 | JK340855 | 176 | _17Z_G11 Bermudagrass Normalized cDNA Library Cynodon dactylon cDNA 5', mRNA sequence. | ES301698.1 | 7.00E-38 |
| Tifway-10-46 | JK340856 | 248 | similar to DHN4 [Hordeum vulgare],LEA protein [Triticum turgidum subsp. durum],[Hordeum vulgare subsp. vulgare] gi|82388|pir|S05546 dehydrin 18 - barley gi|118489|sp|P12949|DH4_HORVU DEHYDRIN DHN4 (B18),dehydrin DHN2 - sorghum (fragment) gi|14..., mRNA sequence. | DN987333.1 | 9.00E-96 |
| Tifway-10-47 | JK340857 | 193 | CFM01-0238 Finger millet developing seed cDNA Eleusine coracana cDNA clone CFM01-0238 5' similar to Best blastX hit, e-value: 1.00E-87, acc.no: AAK27801, ID: 60S ribosomal protein L21,mRNA sequence. | CX264874.1 | 4.00E-41 |
| Tifway-10-48 | JK340858 | 239 | Bermudagrass Normalized cDNA Library Cynodon dactylon cDNA 5' | ES304588.1 | 3.00E-31 |
| Tifway-10-49 | JK340859 | 284 | Bermudagrass Normalized cDNA Library Cynodon dactylon cDNA 5', mRNA sequence. | ES304588.1 | 3.00E-31 |
| Tifway-10-50 | JK340860 | 238 | Bermudagrass cv. Jackpot SSH Library Cynodon dactylon cDNA clone J-88.M13 | BQ825910.1 | 6.00E-66 |
| Tifway-10-51 | JK340861 | 332 | Bermudagrass Normalized cDNA Library Cynodon dactylon cDNA 5' | ES292837.1 | 4.00E-159 |
| Tifway-10-52 | JK340862 | 187 | Bermudagrass Normalized cDNA Library Cynodon dactylon cDNA 5' | ES307394.1 | 2.00E-88 |
| Tifway-10-53 | JK340863 | 280 | Eragrostis curvula cDNA, mRNA sequence. | EH184933.1 | 1.00E-31 |
| Tifway-10-55 | JK340864 | 147 | Bermudagrass Normalized cDNA Library Cynodon dactylon cDNA 5' | ES297499.1 | 5.00E-58 |
| Tifway-10-56 | JK340865 | 147 | Heat Stress SSH cDNA Festuca arundinacea cDNA clone | CK802567.1 | 6.00E-07 |
| Tifway-10-57 | JK340866 | 252 | Zea mays contig45238, mRNA sequence | EZ109579.1 | 1.00E-18 |
| Tifway-10-58 | JK340867 | 269 | Eragrostis curvula cDNA, mRNA sequence. | EH191363.1 | 1.00E-44 |
| Tifway-10-59 | JK340868 | 147 | Bermudagrass Normalized cDNA Library Cynodon dactylon cDNA 5', | ES302487.1 | 2.00E-68 |
| Tifway-10-60 | JK340869 | 306 | Bermudagrass cv. MSU subtracted cold acclimated cDNA library Cynodon dactylon cDNA clone | DN985598.1 | 3.00E-103 |
| Tifway-10-61 | JK340870 | 154 | Bermudagrass Normalized cDNA Library Cynodon dactylon cDNA 5' | ES303408.1 | 1.00E-59 |
| Tifway-10-62 | JK340871 | 282 | Bermudagrass Normalized cDNA Library Cynodon dactylon cDNA 5' | ES306669.1 | 5.00E-81 |
| Tifway-10-64 | JK340872 | 251 | Bermudagrass Normalized cDNA Library Cynodon dactylon cDNA 5', mRNA sequence. | ES307383.1 | 9.00E-102 |
| Tifway-10-65 | JK340873 | 157 | Eragrostis curvula cDNA, mRNA sequence. | EH190380.1 | 4.00E-47 |
| Tifway-10-66 | JK340874 | 186 | Bermudagrass Normalized cDNA Library Cynodon dactylon cDNA 5' | ES306690.1 | 2.00E-58 |
| Tifway-10-67 | JK340875 | 102 | Spartina alterniflora leaf salinity induced expressed sequence tag (EST) Spartina alterniflora cDNA | EH276744.1 | 1.00E-06 |
| Tifway-10-68 | JK340876 | 162 | primary and normalized libraries Pseudoroegneria spicata cDNA clone | FF348486.1 | 5.00E-27 |
| Tifway-10-69 | JK340877 | 262 | Bermudagrass cv. MSU subtracted cold acclimated cDNA library Cynodon dactylon cDNA clone | DN985546.1 | 1.00E-119 |
| Tifway-10-70 | JK340878 | 342 | Bermudagrass Normalized cDNA Library Cynodon dactylon cDNA 5' | ES302790.1 | 1.00E-107 |
| Tifway-10-71 | JK340879 | 397 | Bermudagrass Normalized cDNA Library Cynodon dactylon cDNA 5' | ES294134.1 | 2.00E-94 |
| Tifway-10-72 | JK340880 | 344 | Bermudagrass Normalized cDNA Library Cynodon dactylon cDNA 5' | ES300719.1 | 1.00E-147 |
| Tifway-10-73 | JK340881 | 381 | RTM39 Finger millet pTriplEx2 library 3 Eleusine coracana subsp.africana cDNA 5' | EB086117.1 | 6.00E-18 |
| Tifway-10-74 | JK340882 | 381 | Eragrostis curvula cDNA, mRNA sequence | EH192019.1 | 6.00E-31 |
| Tifway-10-75 | JK340883 | 333 | Bermudagrass Normalized cDNA Library Cynodon dactylon cDNA 5' | ES301946.1 | 9.00E-161 |
| Tifway-10-76 | JK340884 | 363 | Bermudagrass Normalized cDNA Library Cynodon dactylon cDNA 5' | ES298642.1 | 5.00E-88 |
| Tifway-10-77 | JK340885 | 188 | Panicum virgatum early floral buds + reproductive tissue (L) Panicum virgatum cDNA clone CCGF16408 3' | FL790322.1 | 7.00E-06 |
| Tifway-10-78 | JK340886 | 141 | Bermudagrass Normalized cDNA Library Cynodon dactylon cDNA 5' | ES293989.1 | 4.00E-65 |
| Tifway-10-79 | JK340887 | 155 | Bermudagrass Normalized cDNA Library Cynodon dactylon cDNA 5' | ES304948.1 | 1.00E-40 |
| Tifway-10-80 | JK340888 | 168 | Saccharum hybrid cultivar SP80-3280 cDNA clone SCVPRT3087D04 5' | CA269912.1 | 3.00E-10 |
| Tifway-10-81 | JK340889 | 200 | Bermudagrass Normalized cDNA Library Cynodon dactylon cDNA 5' | ES295103.1 | 4.00E-67 |
| Tifway-10-82 | JK340890 | 184 | Bermudagrass Normalized cDNA Library Cynodon dactylon cDNA 5' | ES301548.1 | 1.00E-85 |
| Tifway-10-85 | JK340891 | 156 | Bermudagrass Normalized cDNA Library Cynodon dactylon cDNA 5', mRNA sequence. | ES306510.1 | 4.00E-78 |
| Tifway-10-86 | JK340892 | 92 | no significant homology |  |  |
| Tifway-10-87 | JK340893 | 221 | no significant homology |  |  |
| Tifway-10-88 | JK340894 | 119 | no significant homology |  |  |
| Tifway-10-89 | JK340895 | 188 | no significant homology |  |  |
| Tifway-10-90 | JK340896 | 364 | no significant homology |  |  |
| Tifway-10-91 | JK340897 | 301 | no significant homology |  |  |
| Tifway-10-92 | JK340898 | 231 | no significant homology |  |  |
| Tifway-10-93 | JK340899 | 100 | no significant homology |  |  |
| Tifway-10-94 | JK340900 | 241 | no significant homology |  |  |
| Tifway-10-95 | JK340901 | 145 | no significant homology |  |  |
| Tifway-10-96 | JK340902 | 155 | no significant homology |  |  |
| Tifway-10-97 | JK340903 | 191 | no significant homology |  |  |
| Tifway-10-98 | JK340904 | 252 | no significant homology |  |  |
| Tifway-10-99 | JK340905 | 262 | no significant homology |  |  |
| Tifway-10-100 | JK340906 | 146 | no significant homology |  |  |
| Tifway-10-101 | JK340907 | 189 | no significant homology |  |  |
| Tifway-10-102 | JK340908 | 119 | no significant homology |  |  |
| Tifway-10-103 | JK340909 | 157 | no significant homology |  |  |
| Tifway-10-104 | JK340910 | 123 | no significant homology |  |  |
| Tifway-10-105 | JK340911 | 174 | no significant homology |  |  |
| Tifway-10-106 | JK340912 | 175 | no significant homology |  |  |
| Tifway-10-107 | JK340913 | 342 | no significant homology |  |  |
| Tifway-10-108 | JK340914 | 156 | no significant homology |  |  |
| Tifway-10-109 | JK340915 | 75 | no significant homology |  |  |
|  |  |  |  |  |  |
